# Rapid Steam‐Assisted Temperature Swing Adsorption for Direct Air Capture Using a Rotary Adsorber

**DOI:** 10.1002/advs.202521499

**Published:** 2026-01-25

**Authors:** Junye Wu, Yunhao Chen, Kuihua Wang, Yingjie Huo, Yanlin Chen, Quanwen Pan, Tianshu Ge

**Affiliations:** ^1^ Engineering Research Center of Solar Power & Refrigeration (MOE) Institute of Refrigeration and Cryogenics Shanghai Jiao Tong University Shanghai China; ^2^ Cryogenic Center Hangzhou City University Hangzhou China

**Keywords:** adsorption, carbon capture, rotary adsorber, steam purge, temperature swing

## Abstract

Direct air capture (DAC) of CO_2_ is a key solution for balancing hard‐to‐abate carbon emissions and plays a crucial role in achieving net zero. Common DAC methods typically employ fixed bed reactors packed with granular adsorbents, which suffer from high gas pressure drop, poor heat and mass transfer, and long cycle durations. Additionally, multiple reactors are required for continuous operation, resulting in bulky systems and complex control. This study proposes a novel rotary adsorber‐based DAC strategy, where powdered adsorbents are shaped into structured adsorbents, enabling rapid carbon capture in a single reactor via a steam‐assisted temperature swing adsorption cycle. A ton‐scale‐potential DAC prototype was constructed. Experimental results exhibit a CO_2_ capture rate of 50%–85%, producing high‐purity CO_2_ (>90%) with remarkable CO_2_ productivity of 0.235−0.352 kg_CO2_/kg_adsorbent_/day. A mathematical model was developed to reveal the dynamic variations of key parameters within the rotor. On this basis, optimization strategies were proposed, showing that by implementing heat recovery, the total energy consumption of carbon capture could be reduced to 7.41–9.64 MJ/kg_CO2_. Further enhancement of the adsorbent performance could lower the energy consumption to 2.50–3.14 MJ/kg_CO2_. These findings demonstrate the rotary adsorber's outstanding carbon capture capability, offering an efficient and attractive DAC solution.

## Graphical abstract

This study presents a direct air capture (DAC) device based on a steam‐regeneration rotary adsorber for high‐purity CO_2_. Testing of the prototype demonstrates its capability for continuous and stable carbon capture with the potential to achieve ton‐scale. A mathematical model is established to elucidate the rotary adsorber's operational principles and optimization.

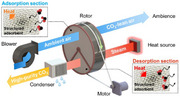

**Abbreviations**
Symbol: Description
*a*
: channel half height
*As*_1_
: specific surface area for the bulk gas
*As*_2_
: specific surface area for the adsorbent
*b*
: channel half width
*C*_CO2_
: CO_2_ concentration
*CRF*
: CO_2_ recovery fraction
*C*_H2O,sat_m: water vapor saturation molar concentration
*C*_inert_
: inert gas concentration
*c*_p_
: constant pressure heat capacity
*C*_T_mol/m^3^
: mol/m^3^total gas molar concentration
*D*_ax_
: axial diffusion coefficient
*DSAR*
: desorption section area ratio
*E*_blower_
: blower energy consumption
*EC*
: CO_2_ capture energy consumption
*E*_comp_
: compressor energy consumption
*E*_elec_
: electrical energy consumption
*E*_motor_
: motor energy consumption
*E*_ther_
: thermal energy consumption
*E*_ther,rec_MJ/kg: thermal energy consumption when adopting heat recovery
*F*_air_m: air flow rate
*F*_des_
: desorption gas flow rate
*F*_steam_
: steam flow rate
*f*_gas_
: ratio of the gas flow channel's cross‐sectional area to the total area
*f*_water_
: water effect factor
*h*
: convective heat transfer coefficient
*h*_steam_
: steam enthalpy
*h*_water_
: kJwater enthalpy
*k*_LDF_
: LDF kinetic coefficient
*L*
: rotor thickness
*m*_ads_
: kadsorbent mass
*M*_CO2_k: kg/mCO_2_ molecular weight
*n*_rot_r/h: rotor rotation speed
*P*_blower_
: blower power
*P*_channel_
: channel perimeter
*p*_CO2_
: CO_2_ partial pressure
*PGC*
: product gas concentration
*P*_motor_
: motor power
*PR*
: CO_2_ productivity
*R*
: ideal gas constant
*rate*_CO2_
: adsorption/desorption rate of CO_2_
*rate*_condense_
: condense rate
*rate*_H2O_
: adsorption/desorption rate of H_2_O
*R*_eq_m: channel equivalent radius
*S*_gas_
: gas flow cross‐sectional area
*Sh*
: Sherwood number
*sto*_CO2_
: stoichiometric ratio of CO_2_ (equal to 1 for CO_2_ and 0 for other gases)
*sto*_H2O_
: stoichiometric ratio of H_2_O (equal to 1 for H_2_O and 0 for other gases)
*S*_total_
: channel total cross‐sectional area
*t*
: operation time
*T*
: thermodynamic temperature
*T*_air_
: air temperature
*T*_gas_
: thermodynamic temperature in the gas side
*Tq*
: motor torque
*T*_sol_
: thermodynamic temperature in the solid side
*T*_steam_
: steam temperature
*v*
: gas velocity
*V*_m_
: ideal gas molar volume
*x*
: molar fraction
*x*_RH_
: relative humidity
*x*_RH,air_
: air relative humidity
*z*
: spatial coordinate of the axis
*δ*
: channel wall thickness
*ΔH*
: heat of adsorption
*λ*
: heat conduct coefficient
*µ*
: dynamic viscosity
*ρ*
: density

## 1 Introduction

Capturing CO_2_ directly from the atmosphere (direct air capture, DAC) plays a critical role in net zero pathways, providing an approach to balance emissions that are difficult to avoid, as well as offering a solution for addressing legacy emissions [1, 2, 3, 4]. In the International Energy Agency's (IEA) Net Zero Emissions (NZE) scenario, DAC technology captures around 980 Mt of CO_2_ in 2050, highlighting its indispensable contribution to climate change mitigation [5, 6]. However, living up to such great expectations requires sustained efforts in advancing DAC to a point where it is energy‐efficient, economical, and scalable for widespread implementation [7, 8].

Adsorption‐based DAC emerges as a promising approach because of its ability to use low‐grade heat for regeneration, non‐corrosive operation, and potential for modular design [1, 9, 10]. Various types of materials have been applied in DAC. According to the different binding mechanisms between CO_2_ and adsorbents, they can be classified into physical adsorbents and chemical adsorbents. Since chemical interactions tend to present higher CO_2_ selectivity at low concentrations, research on chemical adsorbents is currently more extensive than that on physical adsorbents. Chemical adsorbents mainly include two categories: solid alkaline carbonates and amine‐functionalized porous materials. For solid alkaline carbonates, the relatively low adsorption capacity and the need for high desorption temperatures in the absence of additional water lead to substantial energy consumption, which poses challenges for their applications in DAC. Amine‐functionalized porous materials are currently one of the most widely used DAC chemical adsorbents due to high selectivity, high adsorption capacity, and relatively low regeneration temperature. [1]. Nevertheless, there are critical challenges hindering its practical deployment. Given the extremely low concentration of CO_2_ in the air (∼425 ppm), a large volume of air must be processed to capture sufficient CO_2_, yet common adsorbents are prepared in the form of beads or pellets, and they are directly filled in reactors [11, 12, 13]. The dense packing results in substantial pressure drop (>2000 Pa) [14, 15] and thus leads to increased electricity consumption (> 2 MJ/kg_CO2_) for air blowing [16, 17]. To this end, adsorbent shaping techniques have been utilized to transform powder materials into structured adsorbents. Up to now, reported shaping techniques mainly include coating [18], extrusion [19], in situ growth [20], and 3D printing [21]. Among these methods, the coating method is compatible with various types of materials and readily scaled up.

On the other hand, fixed‐bed reactors are commonly used in DAC studies [22, 23, 24]. They are compatible with multiple types of adsorbents, but they cannot operate in a continuous mode unless multiple vessels are utilized in parallel. In this case, careful consideration must be given to coordinate the operation between each vessel, and reasonable control strategies must be designed, which adds to the complexity of the system. Fluidized bed reactors are an alternative to implement DAC [25, 26]. It can operate continuously but imposes strict particle size requirements (typically <100 µm) [27], which limits the adsorbent options. Additionally, it exhibits high adsorbent attrition rates, and the absence of established scale‐up methodologies creates uncertainty in practical applications [28, 29]. While in a moving bed, both the vessel and the adsorbents move together through the adsorption and desorption stages, enabling a continuous process without the need for multiple vessels. However, moving beds have seen limited application in DAC at present. [30, 31, 32, 39]

When it comes to cycle design, temperature vacuum swing adsorption (TVSA) is commonly adopted for current DAC technologies [33]. While this process retains a straightforward and simple operational pattern, it tends to result in elevated CO_2_ partial pressure (>10 kPa) [34, 35, 36] during the desorption phase, leading to a limited desorption driving force and reduced CO_2_ working capacity. Moreover, the prevalent indirect heating/cooling method for temperature swing typically results in insufficient heat transfer and inefficient energy utilization during operation [37]. These factors collectively lead to extended cycle times (> 4 h) [17, 26, 38], low CO_2_ productivity (<0.29 kg_CO2_/kg_adsorbent_/day) [39, 40, 41], and increased heat consumption (>10 MJ/kg_CO2_) [17, 42] in DAC systems.

**SCHEME 1:**
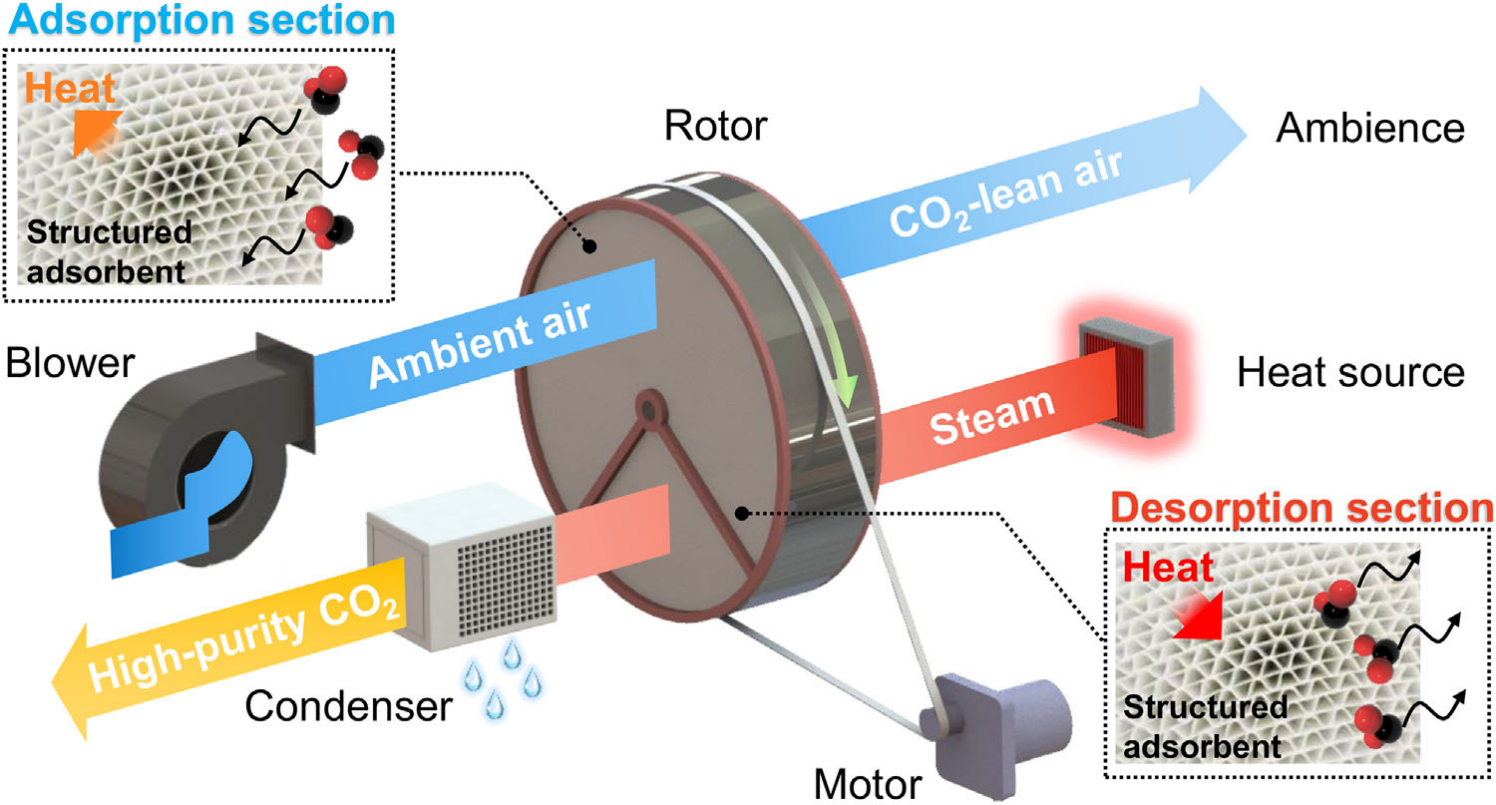
The rotary adsorber‐based direct air capture with steam‐assisted temperature swing adsorption cycle.

To address these challenges, we propose a rotary adsorber‐based DAC strategy (Scheme 1). Using the coating method, we shaped the powdered material into a structured adsorbent. This rotor‐shaped structured adsorbent is packed into a rotating bed reactor and divided into adsorption and desorption sections, which are connected to the adsorption and desorption flow paths, respectively. In the adsorption flow path, ambient air is introduced to contact the adsorbent in the adsorption section, where CO_2_ is captured. In the desorption flow path, steam is injected to contact the adsorbent in the desorption section and heat it up, causing CO_2_ to be desorbed. Subsequently, the desorbed CO_2_ is purified by condensing and separating the steam. During operation, the motor drives the rotor to continuously turn, causing the adsorbent to move constantly between the adsorption and desorption sections, thereby forming a steam‐assisted temperature swing adsorption cycle.

Previously, we proposed an air‐assisted temperature swing adsorption for direct air capture using a rotary adsorber [39], which enables the production of CO_2_‐enriched air suitable for agricultural greenhouses to enhance crop production, exhibiting favorable economic benefits. Similar to the earlier work, in this strategy, due to the optimized geometric configuration of the structured adsorbent (featuring numerous microchannels for airflow), the gas pressure drop is significantly reduced. Furthermore, the rotating bed design allows for continuous adsorption/desorption in a single vessel, resulting in a compact equipment footprint. Additionally, during the desorption stage, the direct contact between steam and adsorbent not only provides the heat required for adsorbent regeneration but also reduces the partial pressure of CO_2_ [34, 43], thereby simultaneously offering both thermal and concentration‐driven desorption incentives to enhance CO_2_ release and enabling rapid cycling. Employing a steam‐assisted temperature swing adsorption, we can produce high‐purity CO_2_ product gas [44] suitable for broader applications, such as carbon sequestration, green fuel synthesis, and chemical production. Steam purge helps improve water management in the DAC process [37, 40], thereby reducing operational energy consumption. Besides, the adsorbents are generated with steam rather than exposed to hot air directly, which has greatly reduced the risk of oxidation.

First, we synthesized a novel type of structured adsorbent and characterized its properties. The results indicate that it not only exhibits favorable adsorption performance but also maintains an airflow pressure drop of no more than 220 Pa at an air velocity of 0–12 m/s. Second, we constructed a rotary adsorber DAC prototype. Through testing under various conditions, we found that it achieves a CO_2_ capture rate of 50%–85%, product gas purity exceeding 90%, while also demonstrating rapid cycling (< 1 h) and high CO_2_ productivity (0.235−0.352 kg_CO2_/kg_adsorbent_/day). Additionally, extended‐term experiments confirmed the stable operation of the rotary adsorber, with a total of 22.03 kg CO_2_ captured during 64 h, equivalent to a capture scale of 2.48 ton_CO2_/year. Third, we developed a mathematical model for a rotary adsorber‐based DAC. Through simulation analysis, we revealed the dynamic changes in key parameters inside the rotor during operation. Using this model, we proposed performance optimization strategies, showing that by implementing heat recovery, the total energy consumption of the rotary adsorber can be reduced to 7.41–9.64 MJ/kg_CO2_. Further enhancement of the adsorbent performance could lower the energy consumption to 2.50–3.14 MJ/kg_CO2_. These findings highlight the efficient carbon capture capability of the rotary adsorber, offering a promising pathway to accelerate the adoption of DAC technology.

## 2 Materials and Methods

### 2.1 Adsorbent Synthesis and Characterizations

We propose an innovative method to prepare a structured adsorbent in the form of a honeycomb. The structured adsorbent (AFHM) has been introduced previously [45]. However, in this study, we fabricate it using a modified version of the method as follows. The liquid mixture of polyethyleneimine (PEI, Gobekie Co. Ltd., branched, Mw≈600) and silica sol (Qingdaohaiyang Co. Ltd., JN‐30, SiO_2_ content 30 wt.%) is used to coat the glass fiber honeycomb (Ecotech Co. Ltd., Foshan, China). Typically, 3.5 kg of PEI was dispersed in 16.5 kg of pure water. After the solution reached equilibrium, 17.5 kg of silica sol was added, and the mixture was stirred for 15 min. The mixture was slowly poured into a glass fiber honeycomb (outer diameter: 800 mm, inner diameter: 60 mm, thickness: 300 mm) to facilitate penetration of the liquid into the honeycomb structure. Unabsorbed liquid draining from the bottom of the honeycomb was collected and reapplied slowly onto the top. This cycle was repeated until the mass of the recovered liquid fell below 10% of the initial mixture. Compressed air was then used to purge the honeycomb, preventing blockage of its gas flow channels by excessive solution. The honeycomb was then dried in an air‐blown thermostatic oven at 80°C for 16 h, yielding the final structured adsorbent (the rotor).

Small pieces of samples (approximately 5 mm×5 mm ×5 mm) were cut from the rotor for characterization tests. Before the tests, samples were degassed under vacuum at 80°C for 5 h. The water adsorption isotherms and water adsorption rates were measured using the DVS Adventure dynamic water vapor sorption analyzer (Surface Measurement Systems). The CO_2_ adsorption isotherms under dry conditions were acquired using the ASAP 2020 (Micromeritics) surface area and porosity analyzer. The CO_2_ adsorption rates were measured using the TGA 8000 (Perkin Elmer). In a typical TGA test, ∼5 mg of sample was loaded into a ceramic crucible and desorbed at 100°C under 90 mL/min of N_2_ for 60 min. The temperature was then adjusted to an adsorption temperature (10°C, 25°C, or 35°C), followed by switching the inlet gas to 90 mL/min of 400 ppm CO_2_ in N_2_ for 120 min. The CO_2_ adsorption under humid conditions was evaluated using a self‐made fixed bed, whose structural details are shown in Figure S1. During the test, 0.6 g of the sample was filled in the column (height: 120 mm, internal diameter: 6 mm). The column was first heated by a temperature control furnace to 100°C followed by a N_2_ purge with a flow rate of 100 mL/min for 60 min. The circulating oil bath was then opened to rapidly cool down the fixed bed to an adsorption temperature (25°C, 50°C, or 75°C). In the adsorption step, mixtures of 0.04%, 0.2%, and 1% CO_2_ in N_2_ (each at a flow rate of 100 mL/min) were passed through the bubbler to carry moisture. The amount of moisture carried was controlled by adjusting the bubbler's temperature. The gas stream was then introduced into the column until the outlet concentrations stabilized. The CO_2_ adsorption capacity was calculated using Equation S1.

### 2.2 Experimental Test of Prototype

A prototype was established to demonstrate the performance of a rotary adsorber‐based DAC. Figure S2 illustrates the process diagram of the prototype, while the corresponding photograph is shown in Figure S3. The structured adsorbent was used as the rotor with a diameter of 800 mm and a thickness of 300 mm. The rotor was divided into an adsorption section and a desorption section, with their cross‐sectional areas occupying 2/3 and 1/6 of the total cross‐sectional area, respectively. The remaining 1/6 of the area was designated as the pressure relief section to enhance the sealing between the adsorption and desorption sections.

The adsorption section is connected to the adsorption flow path, with its inlet drawing ambient air using a centrifugal blower (200−1800 m^3^/h, positive pressure 80−1000 Pa, Suzhou Panli Blower) and its outlet connected to the atmospheric environment. At the inlet and outlet, the air velocity was measured using anemometers (0−10 m/s, accuracy ±3% FS, Xiaoniu Air Velocity), the temperature and humidity were measured using the temperature and humidity sensors (−40–80°C, 0−100% RH, accuracy ±0.5°C, ±3%RH), and the pressure difference was measured using a differential pressure sensor (0−1000 Pa, accuracy ±0.5% FS, Lefoo). Two micropumps (0−1 L/min, 0−40 kPa, Kamoer) were used to extract approximately 1 L/min of air from the inlet and outlet, respectively. The extracted air passed through silica gel drying tubes before entering infrared analyzers (THA100, 0–1000 ppm CO_2_, accuracy ±2% FS, Beijing Taihe Lianchuang) for real‐time CO_2_ concentration measurement.

The desorption section is connected to the desorption flow path, with its inlet receiving steam generated by an electric steam generator (0−320 g/min, flow rate fluctuation ≤ ±2%, rated power of 16 kW). The water supplied to the steam generator is filtered tap water from a water purifier. The desorption flow path outlet is connected to a shell‐and‐tube condenser (heat exchange area of ∼3.5 m^2^). In the condenser, the steam in the desorption outlet gas is condensed using chilled water from a cooling tower as the cooling source. The condensed water and CO_2_ are separated via a U‐shaped bend at the rear end, and the CO_2_ is extracted from the outlet of the U‐shaped bend using a micro pump (F35L‐BX, 0−5 L/min, 0−40 kPa, Chengdu Xinweicheng), and its flow rate was measured using a mass flow meter (0–6 L/min, accuracy ±1.5% FS, Chengde Ferry Automation Equipment). From the outlet of the mass flow meter, approximately 1 L/min of gas is extracted using another micro air pump (0−1 L/min, 0−40 kPa, Kamoer). The gas passes through a silica gel drying tube before entering an infrared analyzer (THA100, 0∼100% CO_2_, accuracy ±2% FS, Beijing Taihe Lianchuang) for real‐time CO_2_ concentration measurement. The temperature at the desorption inlet and outlet was measured using thermal resistors (PT100, −50−300°C, accuracy ±3°C).

During operation, a motor (gear ratio of 2000, Zhejiang Aner) drives the rotor to rotate continuously. The prototype's structural parameters are listed in Table 1. The tests were conducted in Hangzhou from September to October 2024 using the parameters specified in Table 2.

**TABLE 1:** The structural parameters of the steam‐purge rotary adsorber prototype.

Parameter	Value
Rotor outer diameter	800 mm
Rotor inner diameter	60 mm
Rotor thickness	300 mm
Desorption section area ratio	1/6
Adsorption section area ratio	2/3
Channel height	1 mm
Channel width	1.5 mm
Adsorbent wall thickness	0.2 mm
Structured adsorbent mass	42 kg

**TABLE 2:** The operational parameters used in the experimental test of the steam‐purge rotary adsorber prototype. *T*_air_, *x*_RH,air_, *F*_air_, *T*_steam_ experienced fluctuations during the test group 1–11, so the values in the table represent the average value.

Test group	*T*_air_	*x*_RH_ *_,_*_air_	*F*_air_	*n*_rot_	*T*_steam_	*F*_steam_
	°C	%	m^3^/h	r/h	°C	g/min
1^*^	27.7	68	854	1.67	103.3	100
2	26.9	81	498	1.67	105.0	100
3	27.6	74	1228	1.67	103.9	100
4	21.0	52	834	0.73	104.0	100
5	21.8	52	865	1.15	104.0	100
6	28.6	74	847	2.27	106.7	100
7	28.6	73	845	2.96	105.6	100
8	30.1	67	840	1.67	104.9	57
9	30.2	68	842	1.67	105.6	67
10	30.2	68	842	1.67	105.4	72
11	29.5	62	844	1.67	106.6	78
12^**^	22.5−25.9	49−88	829−856	1.67	104.6−108.8	90

* It represents the baseline condition.

** It represents the parameters used in the stability test.

### 2.3 Model Development

For the model development, it is assumed that all gas channels in the rotor have identical specifications. Given that the inlet air and steam conditions are stable, the heat and mass transfer states of channels at the same radial position in the rotor are identical. Furthermore, since the rotor rotates periodically, each channel undergoes the same steam‐assisted temperature swing process at different times. Thus, the behavior of a single channel can represent the state of the entire rotor. Accordingly, we select a single gas channel as the control volume, as shown in Figure S4. In the simulation, the rotor is divided into two sections: adsorption and desorption sections. We list the main assumptions involved in this model and establish the control equations of the model based on the laws of mass and energy balance as elaborated in Section S2.

The mathematical model was implemented in the gPROMS simulation package, and the column pressure was calculated according to the first‐order forward finite difference method (FFDM). The other variables were calculated based on the first‐order backward finite difference method (BFDM). The column length was divided by 20 finite elements. The gas physical properties come from the Multiflash package developed by Infochem Computer Services Ltd.

### 2.4 Calculation of Performance Indicators

For experiments, using the data of temperature, CO_2_ concentration, gas flow rate, and other physical properties, the performance indicators of the steam‐purge rotary adsorber are calculated as follows, where the meaning of the symbols can be found in the Abbreviations table.

The CO_2_ recovery fraction (*CRF*) is calculated by:

(1) $$CRF=\frac{\int_{0}^{t}\left(C_{CO_{2},\mathrm{in},\mathrm{ads}}-C_{CO_{2},\mathrm{out},\mathrm{ads}}\right)dt}{\int_{0}^{t}C_{CO_{2},\mathrm{in},\mathrm{ads}}dt}$$

The product gas concentration (*PGC*), namely the carbon purity, is calculated by:

(2) $$PGC=\frac{\int_{0}^{t}C_{CO_{2},\mathrm{out},\mathrm{des}}dt}{t}$$

The CO_2_ productivity per adsorbent mass (*PR*) is calculated by:

(3) $$PR=\frac{M_{CO_{2}}\int_{0}^{t}\left(C_{CO_{2},\mathrm{out},\mathrm{des}}-C_{CO_{2},\mathrm{in},\mathrm{des}}\right)F_{\mathrm{des}}dt}{V_{m}m_{\mathrm{ads}}t}$$

The energy consumption (*EC*) for the steam‐purge rotary adsorber comprises thermal energy consumption (*E*_ther_) and electrical energy consumption (*E*_elec_):

(4) $$EC=E_{\mathrm{ther}}+E_{\mathrm{elec}}$$

The *E*_ther_ for the steam‐purge rotary adsorber originates from steam generation, and it is calculated by:

(5) $$E_{\mathrm{ther}}=\frac{\int_{0}^{t}\left(h_{\mathrm{steam},T_{\mathrm{des}},p_{\mathrm{des}}}-h_{\mathrm{water},T_{\mathrm{ambient}},p_{\mathrm{ambient}}}\right)F_{\mathrm{steam}}dt}{M_{CO_{2}}\int_{0}^{t}C_{CO_{2},\mathrm{out},\mathrm{des}}F_{\mathrm{des}}dt}$$

The *E*_elec_ for the steam‐purge rotary adsorber includes the energy consumption of the blower and the motor:

(6) $$E_{\mathrm{elec}}=\frac{E_{\mathrm{blower}}+E_{\mathrm{motor}}}{M_{CO_{2}}\int_{0}^{t}\left(C_{CO_{2},\mathrm{out},\mathrm{des}}-C_{CO_{2},\mathrm{in},\mathrm{des}}\right)F_{\mathrm{des}}dt}$$

The blower energy consumption is calculated by (no blower is needed for the desorption flow path):

(7) $$E_{\mathrm{blower}}=P_{\mathrm{blower}}t$$

The motor energy consumption is calculated by:

(8) $$E_{\mathrm{motor}}=P_{\mathrm{motor}}t$$

For the modelling study, all indicators are determined using the same equations as those in experiments, except for those for *PGC, E*_blower_, and *E*_motor_. In the modelling study, it is assumed that the water in the product gas can be completely removed by condensation, so the *PGC* is calculated by:

(9) $$PGC=\frac{\int_{0}^{t}C_{CO_{2},\mathrm{des},\mathrm{out}}F_{\mathrm{steam}}dt}{\int_{0}^{t}\left(C_{CO_{2},\mathrm{des},\mathrm{out}}+C_{\mathrm{inert},\mathrm{des},\mathrm{out}}\right)F_{\mathrm{steam}}+C_{\mathrm{inert},\mathrm{ads},\mathrm{out}}F_{\mathrm{leak}}dt}$$

In the modelling study, the blower power was estimated using the Hagen–Poiseuille equation [5], so the blower energy consumption is calculated by:

(10) $$E_{\mathrm{blower}}=\frac{8L\mu_{\mathrm{air}}v_{\mathrm{air}}}{R_{\mathrm{eq}}^{2}}F_{\mathrm{air}}t$$

In the modelling study, the motor power was estimated based on torque and rotational speed [46]. Consequently, the motor energy consumption in the model is calculated by:

(11) $$E_{\mathrm{motor}}=\frac{2\pi n_{rot}T_{q}}{60}t$$

## 3 Results and Discussion

### 3.1 Characterization of Adsorbent

**FIGURE 1:**
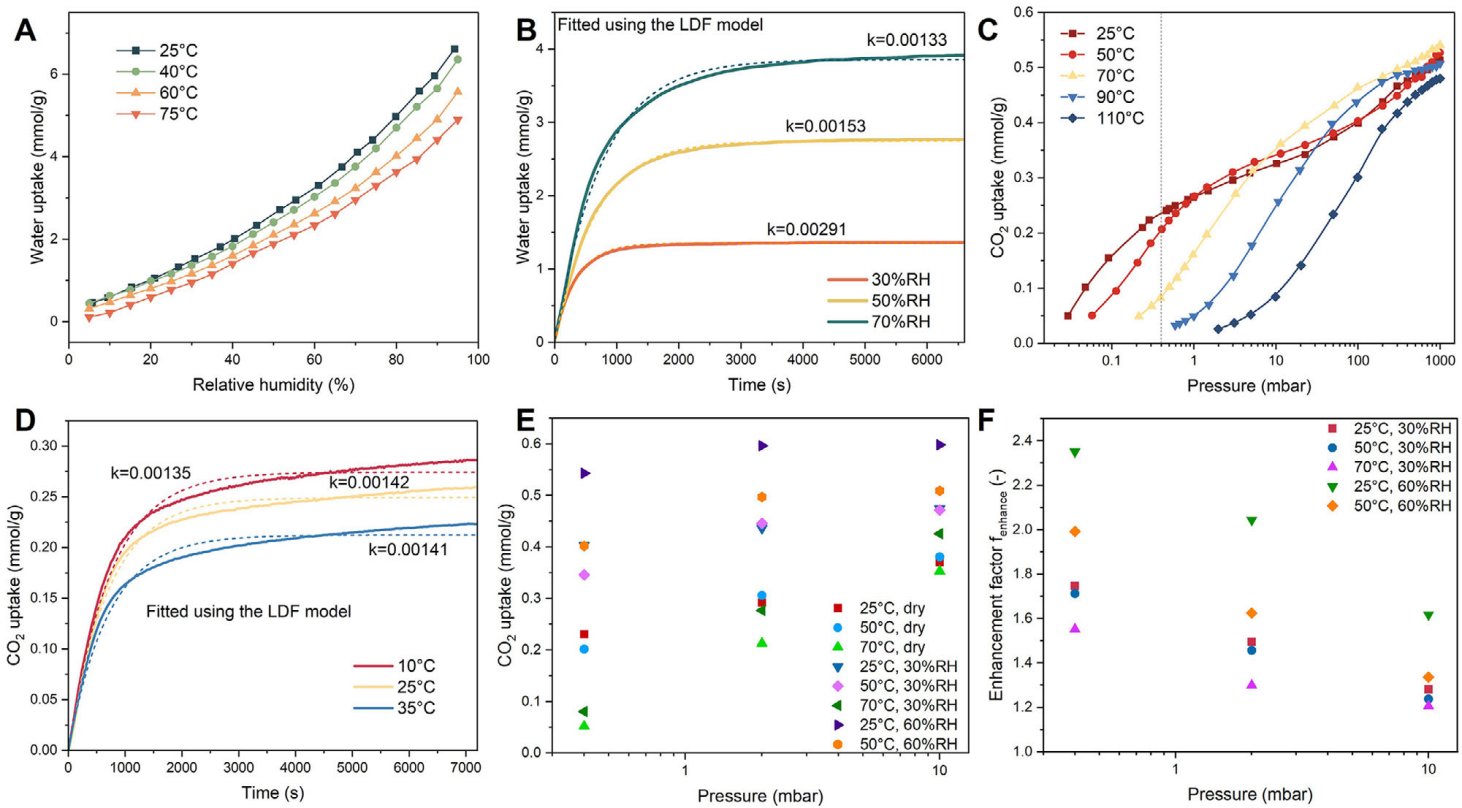
Adsorption performance of the structured adsorbent. (A) Water adsorption isotherms. (B) Dynamic water adsorption curves. The solid lines represent experimental data, while dashed lines represent fitted data. (C) CO_2_ adsorption isotherms under dry conditions. (D) Dynamic CO_2_ adsorption curves. The solid lines represent experimental data, while dashed lines represent fitted data. (E) CO_2_ adsorption isotherms under different relative humidities. (F) Enhancement factors under different relative humidities.

Figure 1A presents the water isotherms of the structured adsorbent at different temperatures. Obviously, the relative humidity (RH) has the primary impact on water uptake, while the temperature has a less significant effect. In a typical range of RH (20%–80%), its water uptake was 0.5–5 mmol/g. Figure 1B shows the dynamic water adsorption curves at 25°C under different RH, which were fitted using the linear driving force (LDF) model [47]. With increasing RH, the adsorption rate decreased (as evidenced by lower kinetic coefficient *k* values), requiring 10–22 min to reach 80% of saturation capacity. The CO_2_ isotherms under dry conditions (Figure 1C) indicate that at 25°C and 0.4 mbar (corresponding to the CO_2_ partial pressure in the air), the CO_2_ uptake was approximately 0.24 mmol/g. When the pressure increased to 1000 mbar, the uptake rose to 0.53 mmol/g. With increasing temperature, the isotherms shifted to the right. Thus, the uptake at 0.4 mbar decreased, while the uptake at 1000 mbar first increased, then decreased. The dynamic CO_2_ adsorption curves (fitted using the LDF model [41]) in 400 ppm CO_2_ (Figure 1D) show that at different temperatures, AFHM exhibited fast adsorption rates, achieving 80% of saturation capacity within 25 min. Besides, a lower temperature resulted in higher uptake, consistent with the patterns observed in the isotherms. Comparable adsorption kinetic coefficients were observed at different temperatures.

The CO_2_ isotherms under different RH measured using the fixed bed are shown in Figure 1E. Under dry conditions, the CO_2_ uptake was largely consistent with that measured using the volumetric method (Figure 1C), indicating the effectiveness of the tests. The uptake under humid conditions was all higher than that under dry conditions, indicating the presence of water promotes CO_2_ adsorption. Therefore, we used a water effect factor *f*_water_ (defined as the ratio of uptake under humid conditions to that under dry conditions at the same temperature) to describe the water effect. As shown in Figure 1F, at 25°C and 30%RH, *f*_water_ was in the range of 1.3–1.8, while at 25°C and 60%RH, *f*_water_ increased to 1.6–2.4. At 50°C, a similar pattern was observed: increasing RH led to a greater promotion effect. At the same RH, increasing temperature resulted in a smaller *f*_water_. Moreover, increasing CO_2_ pressure also led to a smaller *f*_water_, indicating a less significant promotion effect, which is consistent with the results reported by Young et al. [49].

Additionally, we employed a structured adsorbent with a diameter of 800 mm and a thickness of 300 mm to measure the air pressure drop before and after passing through the adsorbent. The results are shown in Figure S5. Within the air velocity range of 0–12 m/s, the airflow pressure drop did not exceed 220 Pa, which is significantly lower compared to that of granular adsorbents (>2000 Pa) [15]. This demonstrates the advantage of structured adsorbents. The results above provide insights into the equilibrium and dynamic adsorption performance of the adsorbent, as well as an evaluation of its airflow resistance. This lays the foundation for our development of a rotary adsorber‐based DAC strategy.

### 3.2 Experimental Investigation of the Rotary Adsorber

#### 3.2.1 Steady State of Rotary Adsorber

**FIGURE 2:**
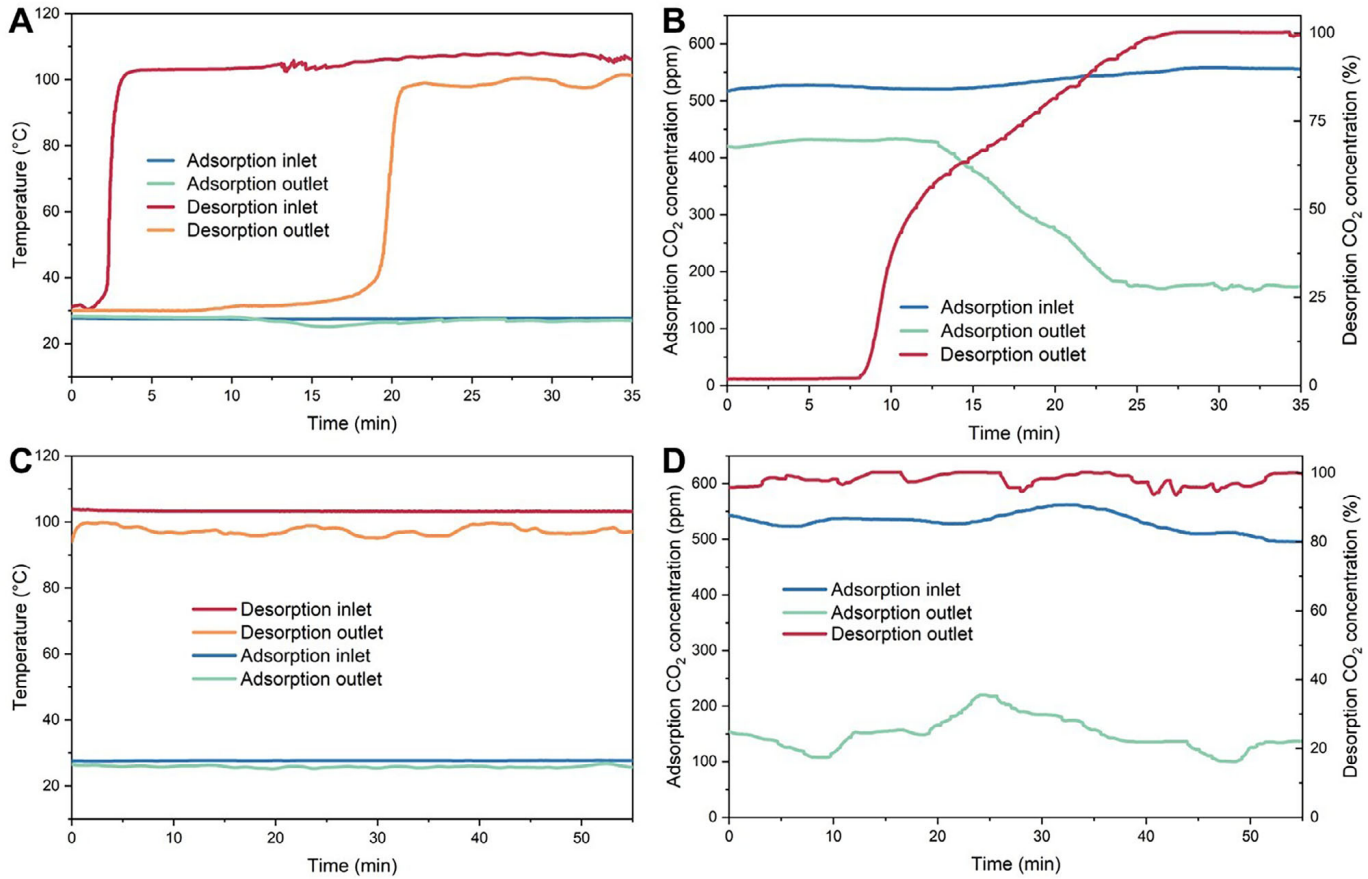
Experimental performance of the rotary adsorber. (A, B) Temperatures and CO_2_ concentrations at the inlet and outlet of flow paths during the unsteady state operation. (C, D) Temperatures and CO_2_ concentrations at the inlet and outlet of flow paths during the steady state operation.

Before the experiment began, the adsorbent in the rotary adsorber was in equilibrium with the air. Under the baseline condition, the unsteady‐state operation at the start of the experiment is shown in Figure 2A, B; Figure S6A. The motor drove the adsorbent to rotate, the blower in the adsorption flow path was activated, and the steam generator began injecting steam into the desorption flow path. As a result, the temperature of the pipes in the desorption flow path and the adsorbent in the desorption section started to rise, causing CO_2_ to gradually desorb and be carried away by the steam. The RH at the adsorption outlet gradually increased. In the condenser, the steam condensed into liquid water, while CO_2_ gradually accumulated and expelled the air. This led to an increase in the CO_2_ concentration at the outlet of the desorption flow path.

Subsequently, the adsorbent moved to the adsorption section, where it came into contact with flowing air. This caused its temperature to decrease, allowing it to adsorb CO_2_ from the air and resulting in a decrease in the CO_2_ concentration at the outlet of the adsorption flow path. Then, the adsorbent moved back to the desorption section, where it again came into contact with steam, heated up, and desorbed, completing a temperature‐swing cycle. After approximately 30 min, the rotary adsorber reached a dynamic equilibrium state, and the outlet temperature, humidity, and CO_2_ concentration in both the adsorption and desorption flow paths stabilized.

Under the baseline condition, the parameter variations of the rotary adsorber at steady state (dynamic equilibrium) are illustrated in Figure 2C, D; Figure S6B. The desorption inlet temperature stabilized at 103°C, while the desorption outlet temperature remained at approximately 97°C. The temperature drop in the desorption flow path is attributed to heat transfer from the steam to the adsorbent, piping, and the environment. The air velocity at the adsorption inlet stabilized at around 7.2 m/s, with a pipe cross‐sectional area of ∼398 cm^2^, corresponding to an air flow rate of 1032 m^3^/h. The RH at the inlet stabilized at 68%, while the RH at the outlet increased to around 80%. This increase can be attributed to two factors: First, the pressure in the desorption flow path was higher than that in the adsorption flow path, and the dynamic sealing between the adsorption and desorption sections was not complete, allowing some vapor leakage. Second, the material adsorbed a significant amount of water in the desorption section, which was later released into the air upon transitioning to the adsorption section (see Section 3.3.2). The adsorption inlet temperature was at the ambient level of 28°C, with the outlet temperature slightly dropping to 26°C, likely due to the evaporative cooling effect of water at the adsorption outlet.

The CO_2_ concentration at the adsorption inlet was approximately 530 ppm, higher than typical outdoor atmospheric levels due to the relatively enclosed laboratory environment where the tests were conducted. Fluctuations in CO_2_ concentrations at both the adsorption and desorption outlets were observed, likely caused by the rotor's continuous rotation and potential inhomogeneity in the adsorbent's properties at different positions. The average CO_2_ concentration at the adsorption outlet was 149 ppm, indicating over 70% of CO_2_ in the air was captured. Meanwhile, the desorption outlet achieved an average CO_2_ concentration of 98%, demonstrating high product gas purity.

#### 3.2.2 Performance Under Varied Conditions

**FIGURE 3:**
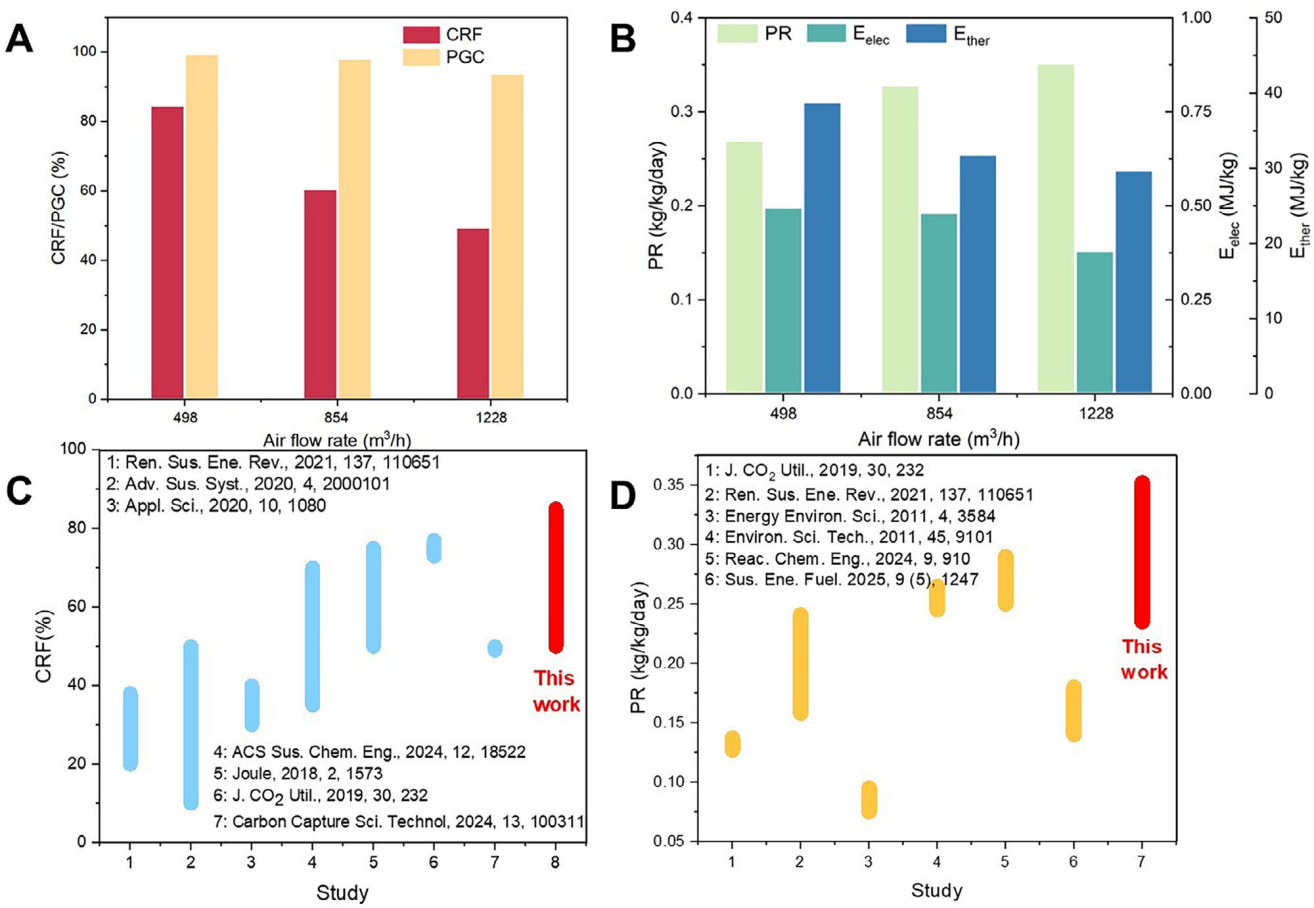
Parametric study of the rotary adsorber. (A, B) Performance indicators under different air flow rates. (C) Comparison of *CRF* and *PR* with other DAC studies.

We adjusted the operating parameters and calculated performance evaluation indicators to examine the rotary adsorber's performance under different conditions. First, we investigated the effect of air flow rate. As shown in Figure 3A, B, as the air flow rate increased, the *CRF*, *PGC*, *E*_elec_, and *E*_ther_ all decreased, while the *PR* increased. This occurs because, at higher flow rates, the rotary adsorber captures CO_2_ less thoroughly, albeit with a higher total amount of CO_2_ captured. Moreover, due to the rotational nature of the rotary adsorber, its internal structure employs dynamic sealing rather than a fully airtight seal. As a result, some gas leakage occurs between the adsorption and desorption sections. The leakage rate depends on the pressure difference between these two flow paths. When the air flow rate increased, the pressure of the air in the adsorption flow path rose, amplifying the leakage and ultimately reducing the *PGC*.

Subsequently, we further evaluated the effects of rotor rotation speed and steam flow rate, and the results are presented in Figure S7. The performance of the rotary adsorber at different rotation speeds is illustrated in Figure S7A, B. As the rotation speed increases, the *CRF*, *PGC*, and *PR* initially rose and then declined, peaking at 1.15 revolutions per hour (r/h). Conversely, both *E*_elec_ and *E*_ther_ first decreased and then increased, reaching their minimum values at approximately 1.15 r/h. Adjusting the rotation speed alters the cycle period, thereby changing the total duration of adsorption and desorption. It the cycle period is too long, the adsorbent, having reached saturation and weakened in adsorption capacity, cannot promptly enter the desorption section. Similarly, a fully desorbed adsorbent cannot return to the adsorption section, reducing adsorbent utilization efficiency. Conversely, if the cycle period is too short, the adsorbent may enter the desorption section before fully exerting its adsorption capacity or return to adsorption before being adequately desorbed, again limiting its effectiveness. Thus, an optimal rotation speed exists. The results suggest that this optimal speed lies between 1.15 and 1.67 r/h, corresponding to a cycle time of 35 to 52 min and an adsorption time of 23 to 35 min. Notably, the adsorbent exhibits its fastest adsorption rate within the first 30 min (Figure 1D), after which the rate gradually declines. This suggests that switching its state around this time may be most efficient, which aligns with the optimal rotation speed observed in the prototype.

The performance of the rotary adsorber under different steam flow rates is illustrated in Figure S7C, D. Increasing the steam flow rate improved the *CRF*, *PGC*, and *PR* while reducing *E*_elec_. *E*_ther_ showed an initial decrease followed by an increase. This behavior occurs because higher steam flow rates enable faster and more complete desorption, which increases the CO_2_ concentration in the product gas stream and enhances the adsorbent's CO_2_ capture capacity. However, higher steam flow rates require greater thermal energy for steam generation, suggesting an optimal flow rate exists. In this experimental setup, the optimal value was approximately 78 g/min. It was observed that during prototype testing, relatively high steam flow rates were required, which resulted in relatively high thermal energy consumption (>22 MJ/kg_CO2_) as mentioned above. This can be attributed to several factors: First, a portion of the heat was used to warm the metal piping, while imperfect thermal insulation led to heat losses through thermal dissipation. Furthermore, due to the pressure difference between the higher‐pressure steam in the desorption flow path and the lower‐pressure air in the adsorption flow path, combined with imperfect sealing of the rotary adsorber, steam leaked into the adsorption flow path during operation, contributing to additional heat losses. Moreover, the steam from the desorption outlet is directly sent to the condenser, resulting in unrecovered heat and additional energy losses. In the model section, we will demonstrate that by improving insulation, reducing leakage, and implementing heat recovery, the thermal energy consumption of the rotary adsorber can be significantly reduced.

Overall, the rotary adsorber demonstrated excellent carbon capture capability, achieving a *CRF* of 50−85%, *PGC* of 70−99%, and *PR* of 0.235−0.352 kg_CO2_/kg_adsorbent_/day. Compared with existing DAC technologies (Figure 3C, D), the rotary adsorber exhibits superior performance, delivering higher carbon capture rates while achieving the highest CO_2_ productivity reported in the literature to date.

#### 3.2.3 Extended‐Term Operation

**FIGURE 4:**
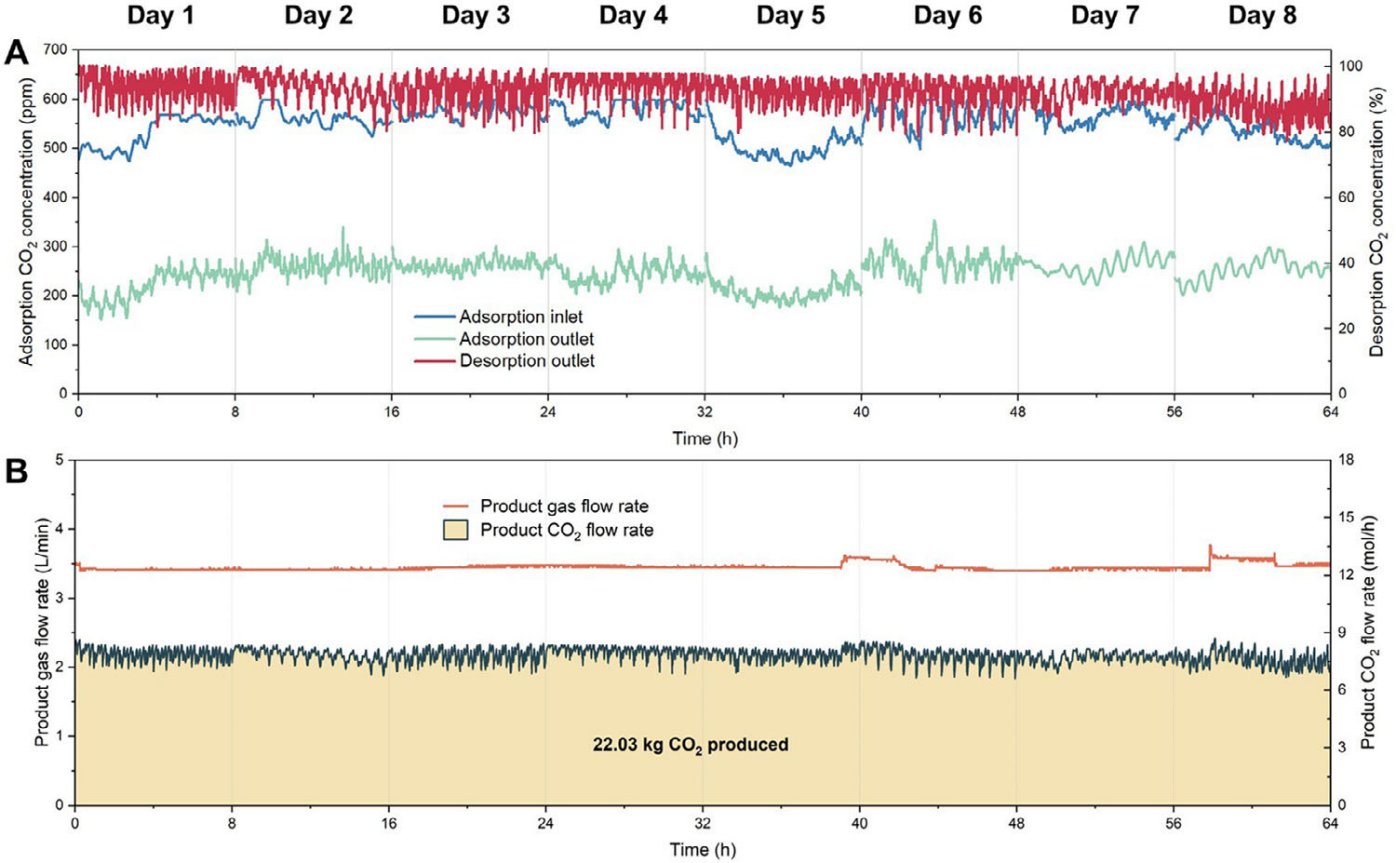
The stability test of the rotary adsorber. (A) The CO_2_ concentration and (B) the temperature variations during the eight‐day tests. (C, E) The daily average *CRF*, *PGC*, and *PR* of the rotary adsorber during the eight‐day tests.

Based on the above investigations, we further conducted an extended‐term test. The experiment began on October 5 and lasted for eight days, with 8 h of testing per day. The variations in CO_2_ concentration and product gas flow rate are shown in Figure 4, while the temperature, RH, and calculated daily average indicators are presented in Figure S8. The results show that the temperature of the air at the adsorption inlet ranged between 20–25°C, while the adsorption outlet temperature was very close to that of the inlet. The desorption inlet temperature was between 100–110°C, and the outlet temperature remained between 90–100°C. The RH of the air at the inlet ranged between 60−85%, while that at the outlet was 65−90%. The CO_2_ concentration at the adsorption inlet ranged between 450–600 ppm, while at the outlet, it was between 150–300 ppm. The CO_2_ concentration at the desorption outlet was between 80%–99%, and the product gas flow rate was maintained at ∼3.4 L/min.

Based on the above data, the performance indicators were calculated. The daily average indicators on the first day are *CRF* of 59%, *PGC* of 94%, and *PR* of 0.263 kg_CO2_/kg_adsorbent_/day, while those on the eighth day are *CRF* of 53%, *PGC* of 88%, and *PR* of 0.246 kg_CO2_/kg_adsorbent_/day. These results demonstrate the stable operation of the equipment. Furthermore, based on the product gas flow rate and CO_2_ concentration, the produced CO_2_ flow rate was calculated to be approximately 7.82 mol/h (using the ideal gas molar volume of 24.5 L/mol at 25°C and 1 bar). By integrating the CO_2_ flow rate curve, the total CO_2_ produced over the 64‐h test period was determined to be 22.03 kg. If the system operates 24 h per day for 330 days per year, the estimated annual CO_2_ capture capacity would reach 2.73 metric tons. These results provide a reference for DAC experimental systems at the ton‐scale‐potential.

### 3.3 Modelling Study of the Rotary Adsorber

#### 3.3.1 Model Validation

Using the isotherm models described in Section 2.3, the fitting results for the CO_2_ and water isotherms are shown in Figure S9A, B. All fitting correlation coefficients (*R* ^2^) exceed 0.96, indicating that these models effectively describe the adsorption characteristics of the materials for CO_2_ and water. Furthermore, we compared the data generated by the model with the CO_2_ breakthrough curves measured using the fixed bed shown in Figure S1. The results are presented in Figure S9C. It can be observed that under different temperatures and humidity levels, the simulated breakthrough curves closely match the experimental ones, with error not exceeding 4%. On this basis, the model was used to simulate the carbon capture performance of the rotary adsorber. As shown in Figure S9D, for the 11 test groups listed in Table 2, the error between the experimental and simulated values of the outlet CO_2_ concentration during the steady state phase does not exceed 17%, confirming the model's validity.

#### 3.3.2 The Variation of Parameters

**FIGURE 5:**
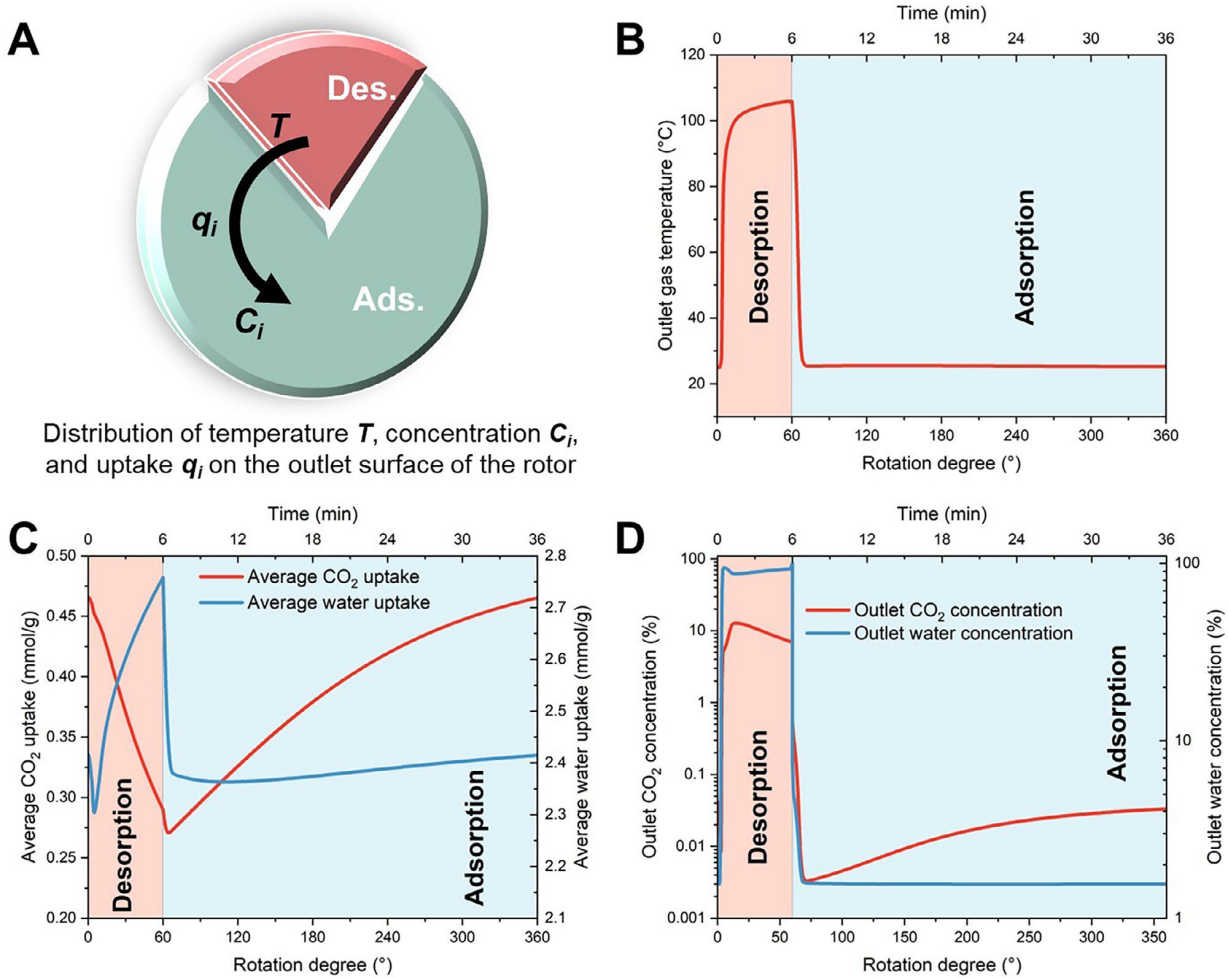
The parameter distribution on the outlet surface of the rotor. (A) The temperature variation. (B) The CO_2_ and water uptake variation. (C) The CO_2_ and water concentration variation.

Using the parameter input listed in Table S3, the temperature, uptake, and concentration distributions on the outlet surface of the rotor in the steady state are shown in Figure 5. The horizontal coordinate of the rotation degree is divided according to the rotation direction of the rotor, as shown in Figure 5A. Note that the gas flow directions for adsorption and desorption are opposite, so the outlets for the adsorption and desorption phases are located on opposite faces of the rotor.

As shown in the desorption section of Figure 5B, when the gas flow channel enters the desorption section, the outlet gas temperature rises sharply, approaching that of the incoming steam. This temperature increase causes a sharp decline in channel's average CO_2_ uptake (indicating CO_2_ desorption) and a rapid rise in outlet CO_2_ concentration, which can be seen respectively from the lines of CO_2_ in the desorption section of Figure 5C, D. As desorption nears completion, the outlet CO_2_ concentration gradually decreases under steam purge as shown in the adsorption section of Figure 5D. When the gas flow channel enters the adsorption section, the outlet gas temperature drops rapidly, eventually stabilizing near ambient air temperature. It's clear from the adsorption section of Figure 5C, D that the channel's average CO_2_ uptake first experiences a very brief decline (due to the high temperature of the adsorbent while the CO_2_ partial pressure rapidly decreases under air purge), followed by a rapid increase (indicating CO_2_ adsorption). This causes the outlet CO_2_ concentration quickly drop to around 0.003 mol.%, after which it slowly recovers until approaching 0.04 mol.%.

Since steam purge is employed during the desorption phase, the temperature rise is accompanied by an increase in water partial pressure, causing the concentration of water at the outlet to reach 80–99 mol.%, while the RH inside the channel also reaches near 100%. Meanwhile, it can be seen from the desorption section of Figure 5C that the channel's average water uptake initially undergoes a brief decline (due to the rapid temperature rise, while the increase in water partial pressure lags slightly), then it rises rapidly. During the adsorption phase, under the effect of air purge, the water concentration at the outlet quickly drops to around 1.7 mol.%, and the RH inside the channel decreases rapidly. As a result, the water uptake declines sharply. Subsequently, as the temperature decreases, the RH inside the channel recovers slightly, leading to a corresponding minor increase in water uptake.

The above results generally indicate that the adsorption‐desorption behaviors of CO_2_ and water exhibit opposite trends. This phenomenon eliminates the need to consume significant additional heat for water desorption during the CO_2_ desorption phase. On the contrary, it provides beneficial effects: the exothermic adsorption of water accelerates adsorbent heating during CO_2_ desorption, while the endothermic desorption of water aids adsorbent cooling during CO_2_ adsorption. This demonstrates the advantage of the steam‐assisted temperature swing cycle.

#### 3.3.3 Parametric Study

The performance of the rotary adsorber is influenced by multiple parameters. Section 3.2.2 has experimentally evaluated the impact of some operational parameters. Herein, we further adjust the structural parameters and environmental parameters and investigate their effects through simulation methods. The results are presented in Figure S10 and Table S4.

It can be seen from Figure S10A that the rotor thickness (namely, the length of the gas flow channel) positively influences *CRF* but negatively impacts *PGC* and *PR*. This is primarily because longer gas channels accommodate a greater amount of adsorbent, enabling more effective capture of CO_2_ from the incoming air. However, longer channels retain a higher volume of non‐condensable gases (N_2_ and O_2_), resulting in a reduction in *PGC*. Furthermore, the increased channel length reduces the utilization rate of the adsorbent per unit mass, leading to a decline in *PR*. From Figure S10B, it is found that the desorption section area ratio (*DSAR*) positively affects *CRF*, *PGC*, and *PR*. An increase in *DSAR* signifies an increase in desorption duration, which facilitates more complete release and recovery of CO_2_. Therefore, more CO_2_ is captured and obtained per cycle, boosting *CRF* and *PR*. In addition, the CO_2_ proportion in the product gas is increased, resulting in improved *PGC*.

Figure S10C shows that air temperature exhibits a negative correlation with the performance of the rotary adsorber, as higher temperatures result in reduced *CRF*, *PGC*, and *PR*. This decline is attributed to the decreased CO_2_ uptake of the adsorbent under low CO_2_ partial pressure and the decline of the adsorbent's performance at elevated temperatures (as outlined in Section 3.1). Figure S10D shows that air humidity positively influences the performance of the rotary adsorber, with higher humidity levels leading to increased *CRF*, *PGC*, and *PR*. This improvement is due to the higher CO_2_ uptake of the adsorbent under high‐humidity conditions (as outlined in Section 3.1). Additionally, in the steam‐assisted CO_2_ desorption phase, water does not extensively desorb, thereby preventing adverse effects observed in common TVSA cycles [37, 50]

Building on the above analysis, we further investigate the relationship between energy consumption and productivity of the rotary adsorber under various conditions. As illustrated in Figure S10E, adjusting all the structural and environmental parameters can enhance *PR* while simultaneously reducing *E*_elec_. This is because when these parameters are varied in a direction that boosts *PR*, they essentially increase the working capacity of the adsorbent without leading to a rise in total electrical energy consumption, thereby reducing the electricity consumption per unit mass of CO_2_ captured. As shown in Figure S10F, adjusting the air temperature and humidity can increase *PR* while decreasing *E*_ther_. This indicates that under suitable environmental conditions, the rotary adsorber can achieve better overall performance without incurring higher energy costs. However, when altering the rotor thickness and *DSAR*, an increase in *PR* is accompanied by a rise in E_ther_, with the impact of changing rotor thickness being more pronounced. The reason is that reducing rotor thickness improves the utilization efficiency of the adsorbent per unit mass, increasing *PR*, but the total mass of CO_2_ captured decreases while the steam flow rate remains unchanged, leading to an increase in *E*_ther_. On the other hand, increasing *DSAR* allows for more thorough desorption of the adsorbent, capturing more CO_2_, but the extended desorption time also results in higher total steam consumption, thus increasing *E*_ther_.

It is observed that in the simulation study, the thermal energy consumption was lower than that in the experiments. This discrepancy is primarily attributed to two reasons. First, the experimental setup was not completely adiabatic, and the pipes have heat capacity, leading to heat dissipation to the environment and metals along the flow paths during operation. Second, the adsorption and desorption sections were not perfectly sealed. To minimize air leakage from the adsorption to the desorption flow path, a higher steam flow rate was adopted, ensuring the pressure in the desorption flow path was no less than that in the adsorption flow path. This resulted in steam leakage from the desorption to the adsorption flow path, causing the actual steam consumption to exceed the required amount for desorption. These issues can be addressed in future work by improving thermal insulation and adopting improved sealing measures. Through the parametric study and a comprehensive consideration of *CRF*, *PGC*, *PR*, and *EC*, it was found that either excessively large or small rotor thickness and *DSAR* values would push performance indicators toward unfavorable results. Therefore, a moderate rotor thickness (∼0.3 m) and *DSAR* (∼1/6) represent a better design choice, which is used in the following optimization research.

#### 3.3.4 Optimization Strategies

**FIGURE 6:**
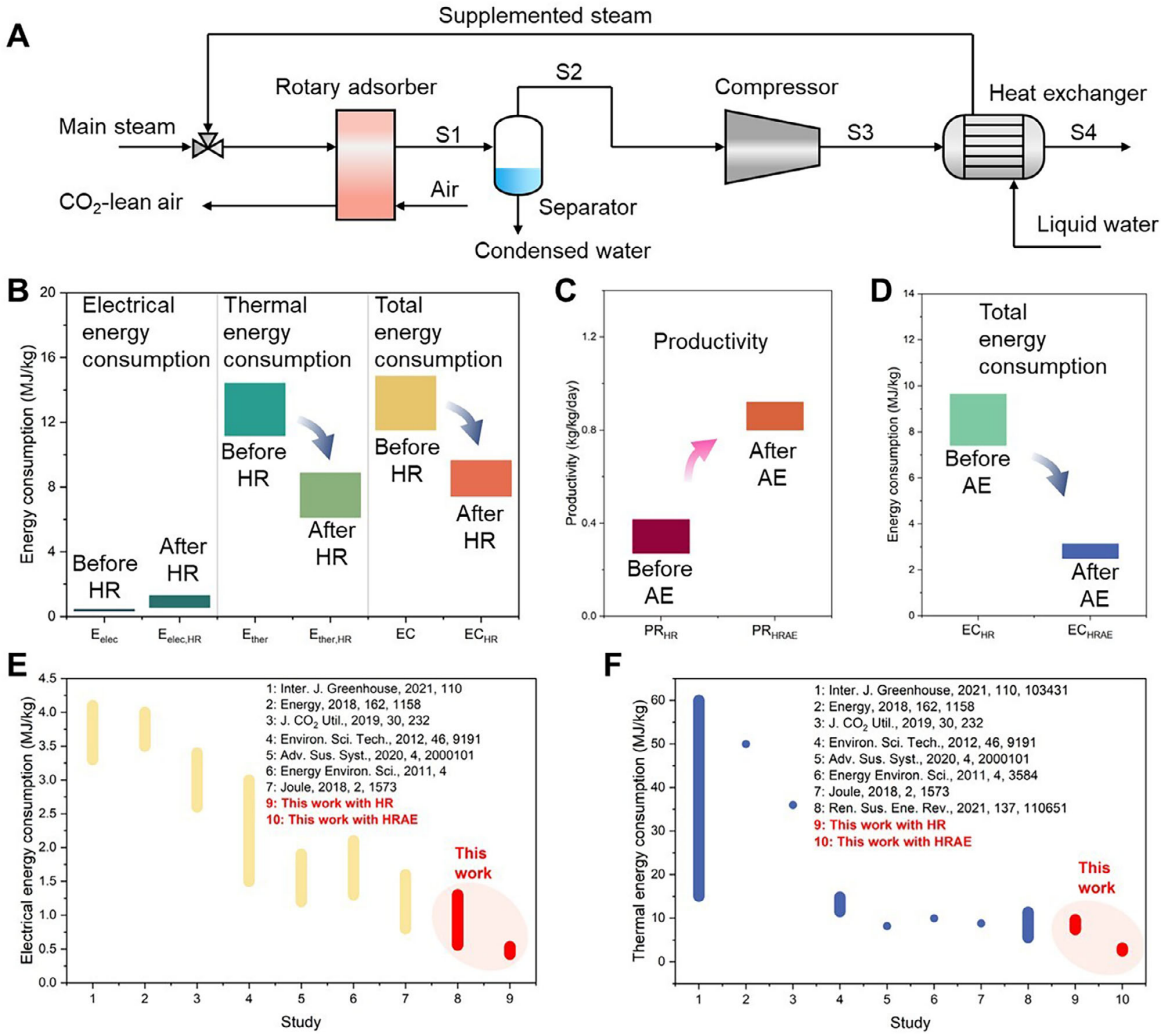
The optimization strategies of the rotary adsorber. (A) The schematic diagram of a heat recovery module. (B) The performance improvement by adopting heat recovery (HR). (C, D) The performance improvement by adopting heat recovery and adsorbent enhancement (HRAE). (D−F) The comparison of thermal and electrical energy consumption with other DAC studies.

Based on the above results, this section proposes strategies to optimize the performance of the rotary adsorber. It is observed that the energy consumption of the rotary adsorber is primarily composed of thermal energy, and the gas exiting the desorption section during operation remains at high temperature and contains untapped thermal energy. Therefore, we propose a heat recovery (HR) strategy to reduce the energy consumption of the rotary adsorber. As shown in Figure 6A, post‐desorption, a stream (S1) comprising steam, condensed steam (water droplets), and CO_2_ is obtained. This stream is directed to a separator to remove the liquid phase water, then the stream (S2) is fed into a compressor to elevate its pressure and temperature, consuming mechanical work in the process. The now heated and compressed vapor stream (S3) is condensed in a heat exchanger, heating incoming liquid water and converting it into steam that is supplemented to the main steam stream. This process allows the utilization of recovered heat to generate a portion of the steam required for desorption purging.

We assume that the compressor's adiabatic efficiency is 0.85, compressing S2 to 3 bar. In the heat exchanger, liquid water enters at 1 bar and 25°C and exits as saturated steam at 105°C, based on which we built a model in Aspen Plus software (Figure S15). Using the input parameters listed in Table S5, we calculate the energy‐saving potential of this heat recovery strategy under different conditions. In group HR1, the average temperature of the desorption outlet gas from the rotary adsorber was 91.4°C, with molar fractions of CO_2_, N_2_, O_2,_ and H_2_O being 8.39%, 0.18%, 0.05%, and 91.39%, respectively. The original thermal energy consumption was 11.68 MJ/kg_CO2_, but with the heat recovery strategy, 1.06 kg of steam per kg CO_2_ captured could be recovered, reducing the thermal energy consumption to 8.86 MJ/kg_CO2_. Additionally, the electrical energy consumption due to compression was only 0.23 MJ/kg_CO2_. The energy‐saving effects for other groups are shown in Figure 6B. With heat recovery, thermal energy consumption decreased to 6.11–8.86 MJ/kg_CO2_ (a reduction of 21%–57%), while electrical energy consumption ranged from 0.56–1.30 MJ/kg_CO2_, and total energy consumption of 7.41–9.64 MJ/kg_CO2_.

Adsorbent is one of the core factors influencing DAC performance. Therefore, based on the aforementioned results, we further explored the carbon capture efficacy of the rotary adsorber with adsorbent enhancement. A structured adsorbent without a substrate presents a promising solution because it avoids the compromise of adsorption capacity resulting from the incorporation of an inert substrate [14]. As shown in Figure S16, our lab has successfully developed a substrate‐free structured adsorbent (PEI‐functionalized silica honeycomb, PEI‐SiO_2_‐HC). This monolith is fabricated using silica powder as the raw material, processed through mold extrusion, sintering, and air‐drying, followed by PEI loading via impregnation. Dynamic and equilibrium CO_2_ adsorption tests revealed that while its CO_2_ adsorption rate is slightly slower than that of AFHM, its saturation capacity is significantly improved. We fitted its adsorption performance using models (Section S7.2) and incorporated these parameters into the rotary adsorber model, conducting simulations with the parameters listed in Table S6. To match its kinetics, we adjusted the rotation speed to 1 r/h and the *DSAR* to 1/8.

As shown in Table S6, after adopting the substrate‐free adsorbent, the productivity of the rotary adsorber reaches 0.800–0.920 kg_CO2_/kg_adsorbent_/day, while the electrical energy consumption decreases to 0.13–0.15 MJ/kg_CO2_, and the thermal energy consumption drops to 3.76–4.50 MJ/kg_CO2_, resulting in a total energy consumption of 3.89–4.65 MJ/kg_CO2_. As shown in Figure 6C, D, when further applying the heat recovery strategy (heat recovery and adsorbent enhancement, HRAE), the electrical energy consumption ranges from 0.425–0.536 MJ/kg_CO2_, and the thermal energy consumption is reduced to 2.06–2.71 MJ/kg_CO2_, yielding a total energy consumption of 2.50–3.14 MJ/kg_CO2_. Such energy consumption levels are significantly lower than those of other DAC studies (Figure 6E, F) and comparable to those of post‐combustion carbon capture (2–3 MJ/kg_CO2_) [51, 52], demonstrating the promising performance of rotary adsorber‐based DAC.

## 4 Conclusions

In this work, we propose a rotary adsorber‐based DAC strategy. It features a steam‐assisted temperature cycle: CO_2_ is captured from ambient air and released under a hot steam purge. We developed structured adsorbents and characterized their CO_2_ adsorption performance under DAC conditions. Based on this, a rotary adsorber DAC prototype was constructed and tested. The results demonstrate its capability for continuous and stable carbon capture. Benefiting from the efficient heat and mass transfer of the structured adsorbent and steam purge temperature swing, it achieves rapid cycling (each adsorption‐desorption cycle takes ∼36 min) and produces CO_2_ with a purity of 98%. Variable‐condition tests investigated the effects of air flow rate, rotation speed, and steam flow rate on its carbon capture performance, showing a CO_2_ capture of 50%–85% and a productivity of 0.293–0.352 kg_CO2_/kg_adsorbent_/day.

Additionally, we developed a mathematical model for the rotary adsorber. By simulating the distribution and dynamics of temperature, concentration, and adsorption capacity within the rotor, we elucidated the intrinsic working mechanism of the rotary adsorber. Building on this, we used the model to conduct studies on structural and environmental parameters, analyzing their impact on the rotary adsorber's performance and revealing the trade‐off between productivity and energy consumption. We propose strategies for further optimizing the rotary adsorber's performance. By recovering heat from the desorption outlet steam, the energy consumption of the rotary adsorber can be reduced to 7.41–9.64 MJ/kg_CO2_. Moreover, by combining adsorbent performance enhancement with heat recovery, the carbon capture energy consumption can be further lowered to 2.50–3.14 MJ/kg_CO2_. These results present a highly attractive DAC solution.

In subsequent work, the rotary adsorber can be integrated with downstream CO_2_ utilization or storage modules to enable zero‐ or negative‐carbon systems. Further techno‐economic analysis and life cycle assessment will be conducted to evaluate the feasibility and sustainability of this rotary adsorber‐based DAC approach, ultimately supporting the transition toward a sustainable energy future while maximizing economic and environmental benefits.

## Author Contributions

J.W. and T.G. did conceptualization. J.W., Y.C., and K.W. did the methodology. J.W., Y.C., and Y.H. did data curation. J.W. and Y.C. did the investigation. J.W. and T.G. did visualization. T.G did the supervision. J.W. did the writing – original draft. J.W., Y.C., K.W., Y.H., and T.G. did Writing – review & editing

## Conflicts of Interest

There are no conflicts of interest.

## Supporting information




**Supporting File**: advs74044‐sup‐0001‐SuppMat.docx.

## Data Availability

The data supporting the findings of this study are available from the corresponding author upon reasonable request.
