# Supplementary material for: Rapid Steam‐Assisted Temperature Swing Adsorption for Direct Air Capture Using a Rotary Adsorber
Source: Adv Sci (Weinh). 2026 Jan 25;13(19):e21499. doi: 10.1002/advs.202521499 (PMC13045267; doi:10.1002/advs.202521499)
Supplement: Supplementary file 1 — Supporting File: advs74044‐sup‐0001‐SuppMat.docx. [file ADVS-13-e21499-s001.docx]

**Supporting Information**

**Rotary adsorber with rapid steam-assisted temperature swing adsorption cycle for direct air capture**

Junye Wu,^†1^ Yunhao Chen,^†1^ Kuihua Wang, ^1^ Yingjie Huo, ^1^ Yanlin Chen,^1^ Quanwen Pan,^2^ and Tianshu Ge*^1^

^1^Engineering Research Center of Solar Power & Refrigeration (MOE), Institute of Refrigeration and Cryogenics, Shanghai Jiao Tong University, Shanghai 200240, China.

^2^ Cryogenic Center, Hangzhou City University, Hangzhou, 310015, China

*Corresponding author: [baby_wo@sjtu.edu.cn](mailto:baby_wo@sjtu.edu.cn) (Tianshu Ge)

^†^These authors contribute equally to this work.

# S1. Experimental methods


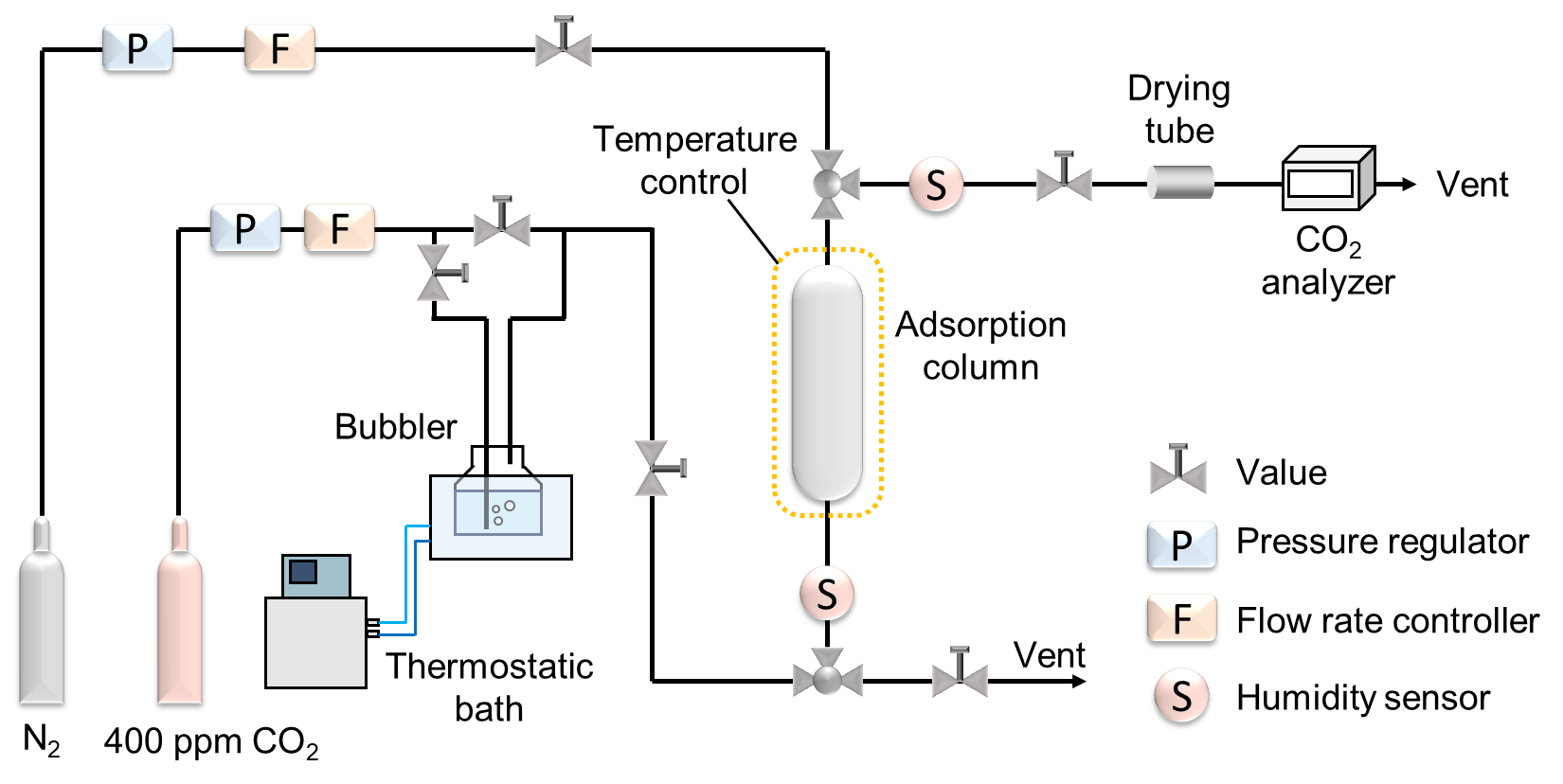


Figure S1 Experimental set up of the self-made fixed bed testing platform.

$q=\frac{\int_{0}^{t} \left(C_{\text{in}}-C_{\text{out}}\right)\times10^{-6}F_{\text{gas}}dt}{V_{\text{m}}m_{\text{ads}}}$ (S1)

*q*: CO_2_ adsorption capacity (mmol/g); *t*: adsorption time (min); *C*_in_: inlet gas CO_2_ concentration (ppm); *C*_out_: outlet gas CO_2_ concentration (ppm); *F*_gas_: gas flow rate (mL/min); *V*_m_: ideal gas molar volume (mL/mmol); *m*_ads_: adsorbent mass (g).


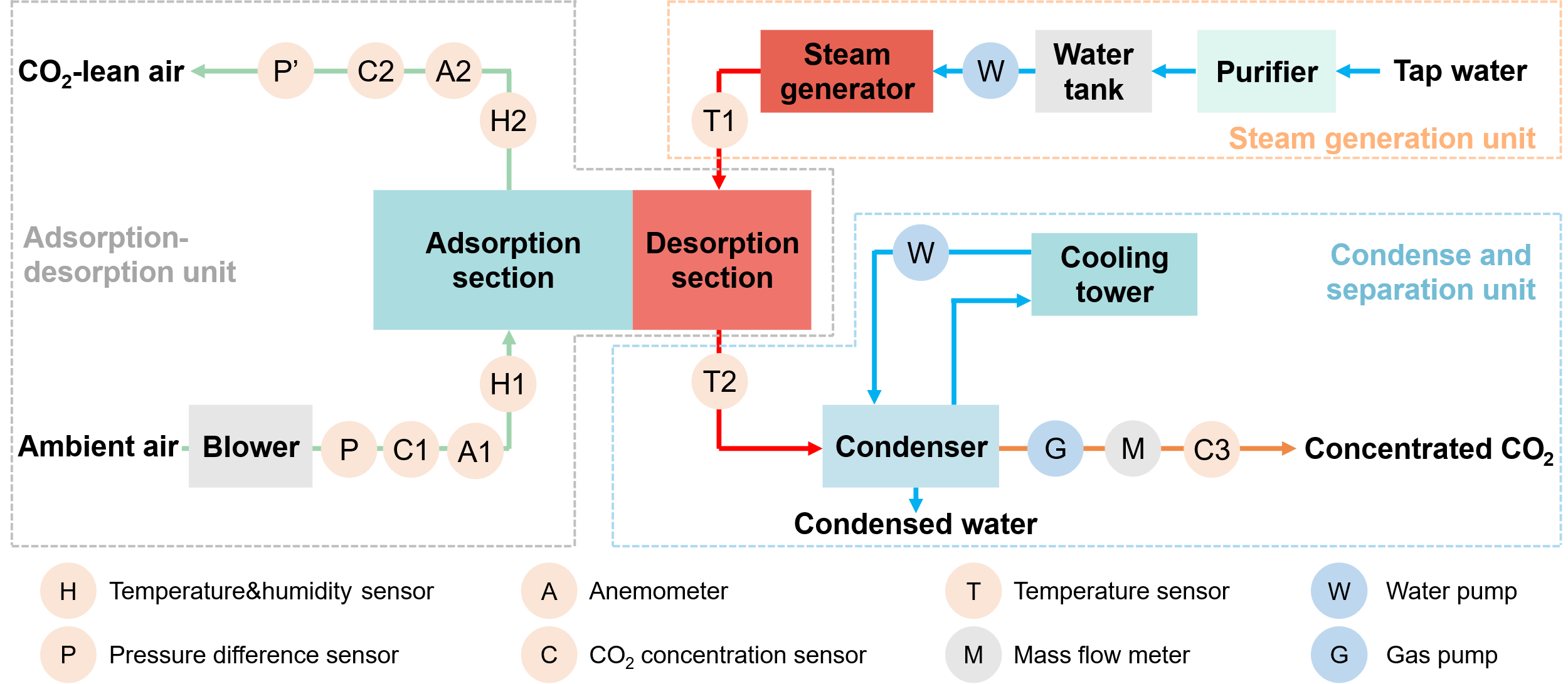


Figure S2 The process diagram of the rotary adsorber prototype. The prototype consists of an adsorption-desorption unit, a steam generation unit, and a condense and separation unit. The locations of the sensors are marked in the diagram. If there are multiple sensors of the same type, they are numbered accordingly. The pressure difference sensors P and P' indicate that the pressure difference between these two points was measured.


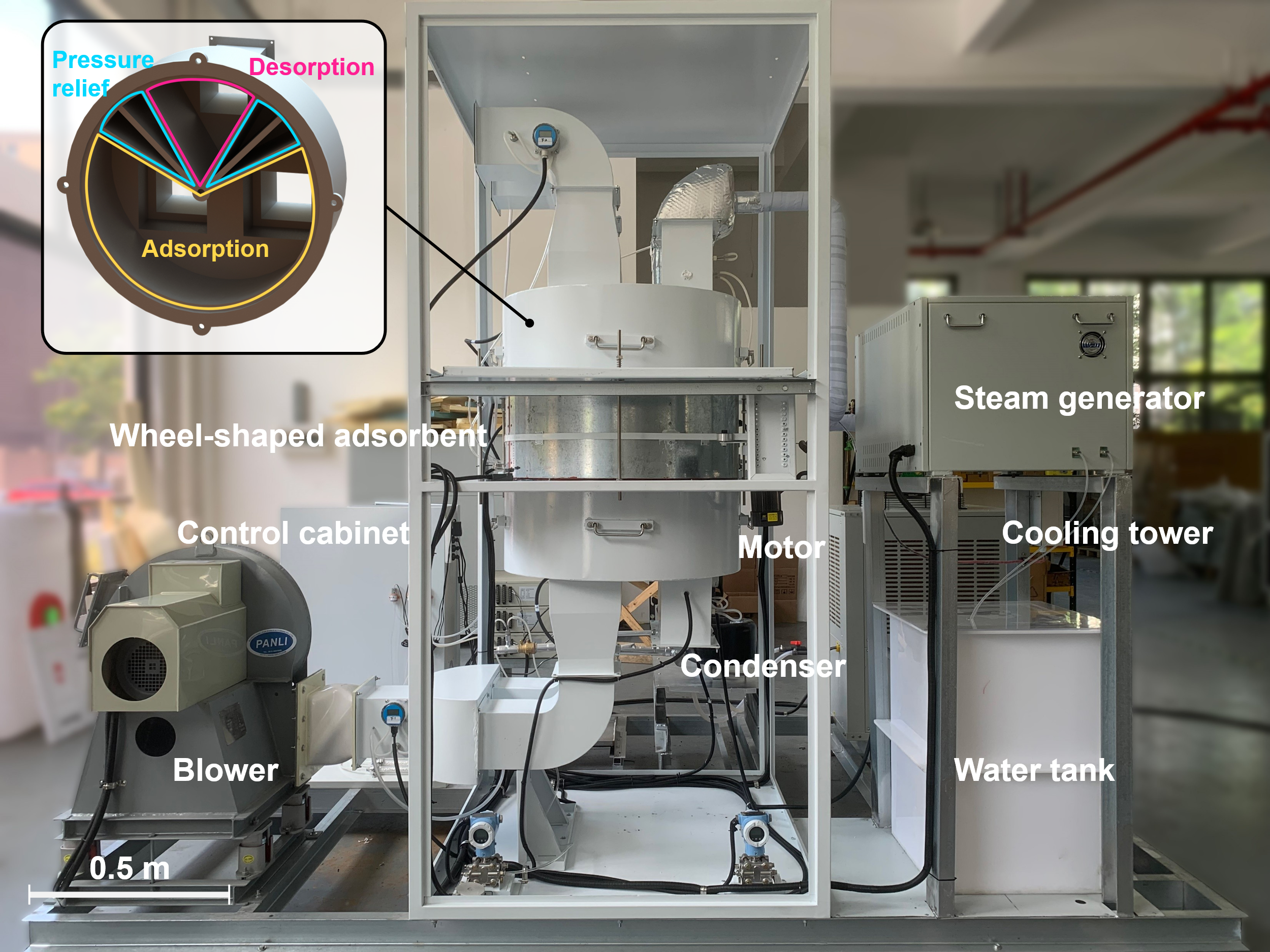


Figure S3 The photograph of the steam-purge rotary adsorber prototype.

# S2. Model development


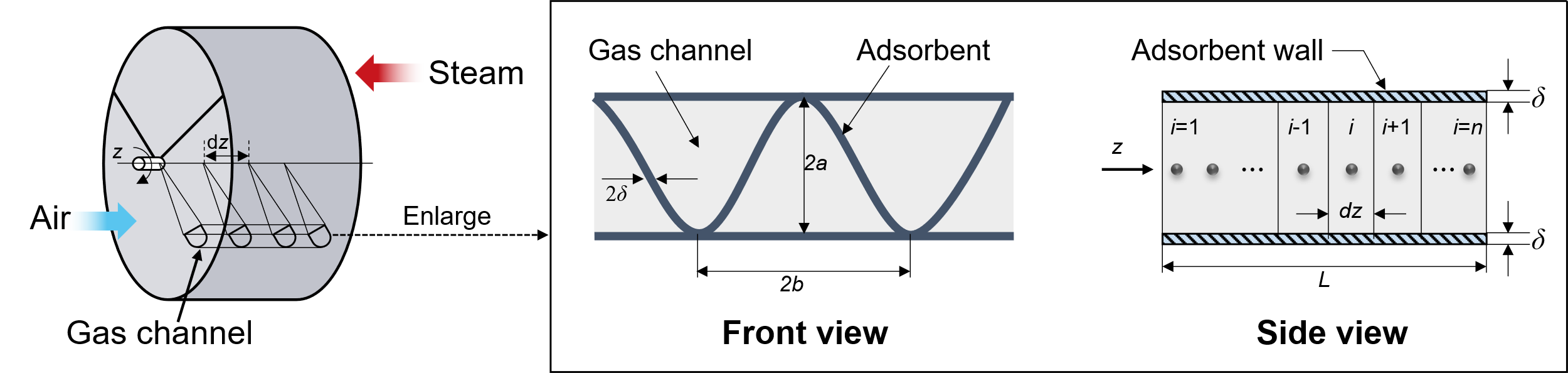


Figure S4 Schematic of the steam-purge rotary adsorber model.

## S2.1 Assumptions

1. All gas channels have identical structures and materials, with adsorbent uniformly distributed.
2. Temperature, pressure, and concentration gradients in the radial direction are neglected.
3. Inlet conditions are spatially uniform but very over time.
4. The heat and mass transfer coefficients between the gas and the adsorbent remain constant throughout the rotor.
5. Heat loss from the channel is negligible during all steps of the cycle.
6. Air is considered to consist of oxygen and nitrogen in addition to CO_2_ and water vapor, and the steam is pure.
7. The ideal gas law and ideal mixture behavior are assumed for non-condensable components.
8. Water adsorption can enhance the adsorption of CO_2_ by supported amines, but CO2 adsorption does not affect water adsorption.
9. During the desorption step, condensed water does not penetrate the adsorbent pores due to the high flow rate of desorbed CO_2_ exiting the pores in the opposite direction. Thus, heat is conducted into the adsorbent and is not transferred by steam diffusion within the adsorbent phase following steam condensation.

## S2.2 Control volume size

As shown in Figure S4, a single gas flow channel is selected as the control volume. From the front view, it can be seen that its cross-section is sinusoidal, with a height of 2*a* and a width of 2*b*. The walls of the gas flow channel consist of adsorbent material, with a thickness of 2*δ*. Based on studies of sinusoidal channels, the channel total cross-sectional area (*S*_total_) is calculated using the formular:

$S_{\text{total}}=2ab$ (S2)

Let the ratio of the gas flow channel's cross-sectional area to the total area be *f*_gas_. Then, the cross-sectional area of the gas flow channel (*S*_gas_) is:

$S_{\text{gas}}=f_{\text{gas}}\cdot S_{total}$ (S3)

The perimeter of the sinusoidal gas flow channel is calculated by:

$P_{\text{channel}}=2b+2\sqrt{b^{2}+\left(a\pi\right)^{2}}\frac{3+\left(2b/a\pi\right)^{2}}{4+\left(2b/a\pi\right)^{2}}$ (S4)

The equivalent radius (*R*_eq_) of the gas flow channel is calculated by:

$R_{\text{eq}}=\frac{2S_{\text{gas}}}{P_{\text{channel}}}$ (S5)

In the side view of the channel, the total length (i.e., the thickness of the rotor core) is denoted as *L*. The channel is discretized into *n* points along the *z*-direction, making the length of each discrete segment *dz*. Since two adjacent gas flow channels share a common wall, the wall thickness of each gas flow channel is *δ*.

## S2.3 Model equations

The equations involved in this model are described below and the meaning of the symbols are illustrated in the Nomenclature table.

(1) Mass balance

$\begin{matrix} \frac{\partial C _{i}}{\partial t}=\frac{As_{2}}{As_{1}}\frac{\partial}{\partial z}\left(D_{\text{ax},i}C_{T}\frac{\partial x_{i}}{\partial z}\right)-\frac{\partial}{\partial z}\left(vC_{i}\right) \\ -\frac{As_{2}}{As_{1}}\rho_{\text{ads}}\left(sto_{\text{CO}_{2}}rate_{\text{CO}_{2}}+sto_{\text{H}_{2}\text{O}}rate_{\text{H}_{2}\text{O}}\right)-\frac{As_{2}}{As_{1}}sto_{\text{H}_{2}\text{O}}rate_{\text{condense}} \end{matrix}$ (S6)

where *i* represent the CO_2_, H_2_O, N_2_, or O_2_.

(2) Energy balance

$\rho_{\text{gas}}c_{\text{p,gas}}\frac{\partial T_{\text{gas}}}{\partial t}=\frac{\partial}{\partial z}\left(\lambda_{\text{gas}}\frac{\partial T_{\text{gas}}}{\partial z}\right)-\rho_{\text{gas}}c_{\text{p,gas}}\frac{\partial}{\partial z}\left(vT_{\text{gas}}\right)+\frac{h}{As_{1}}\left(T_{\text{sol}}-T_{\text{gas}}\right)$ (S7)

$\begin{matrix} \rho_{\text{ads}}c_{\text{p,ads}}\frac{\partial T_{\text{sol}}}{\partial t}=\frac{\partial}{\partial z}\left(\lambda_{\text{ads}}\frac{\partial T_{\text{sol}}}{\partial z}\right)+\frac{h}{As_{2}}\left(T_{\text{gas}}-T_{\text{sol}}\right) \\ +\rho_{\text{ads}}\left(\Delta H_{\text{CO}_{2}}rate_{\text{CO}_{2}}+\Delta H_{\text{H}_{2}\text{O}}rate_{\text{H}_{2}\text{O}}\right)+L_{\text{H}_{2}\text{O}}rate_{\text{condense}} \end{matrix}$ (S8)

(3) Adsorption isotherm and kinetics

For the modified AFHM used in the steam-purge rotary adsorber, its water adsorption isotherm is fitted using the temperature-dependent GAB model[1]:

$q_{\text{H}_{2}\text{O}}=c_{\text{m,GAB}}\frac{c_{\text{G},T}K_{\text{ads,}T}x_{\text{RH}}}{\left(1-K_{\text{ads},T}x_{\text{RH}}\right)\left(1+\left(c_{\text{G},T}-1\right)K_{\text{ads},T}x_{\text{RH}}\right)}$ (S9)

where *c*_m,GAB_ is fitted parameter, while *K*_ads,_*_T_* and *c*_G,_*_T_* are temperature-dependent parameters and fitted using the empirical formulas:

$c_{\text{G},T}=c_{\text{G}1}-c_{\text{G}2}\cdot c_{\text{G}3}^{T}$ (S10)

$K_{\text{ads},T}=K_{\text{ads}1}-K_{\text{ads}2}\cdot K_{\text{ads}3}^{T}$ (S11)

For the modified AFHM, its CO_2_ adsorption isotherm under the dry condition is fitted using the dual-site Toth model[2]:

$q_{\text{CO}_{2}\text{,dry}}=\frac{n_{\text{s,}T,1}b_{T,1}p_{\text{CO}_{2}}}{\left(1+\left(b_{T,1}p_{\text{CO}_{2}}\right)^{t_{T,1}}\right)^{1/t_{T,1}}}+\frac{n_{\text{s,}T,2}b_{T,2}p_{\text{CO}_{2}}}{\left(1+\left(b_{T,2}p_{\text{CO}_{2}}\right)^{t_{T,2}}\right)^{1/t_{T,2}}}$ (S12)

where *n*_s,_*_T_*_,1_, *b_T_*_,1_, *t_T_*_,1_, *n*_s,_*_T_*_,2_, *b_T_*_,2_, *t_T_*_,2_ are temperature-dependent parameters and fitted using the empirical formulas:

$n_{s,T,1}=n_{s,T,1,1}+n_{s,T,1,2}T+n_{s,T,1,3}T^{2}$ (S13)

$b_{T,1}=b_{T,1,1}-b_{T,1,2}b_{T,1,3}^{T}$ (S14)

$t_{T,1}=t_{T,1,1}+t_{T,1,2}T^{t_{T,1,3}}$ (S15)

$n_{s,T,2}=n_{s,T,2,1}\left(T-n_{s,T,2,2}\right)$ (S16)

$b_{T,2}=b_{T,2,1}T^{b_{T,2,2}}$ (S17)

$t_{T,2}=t_{T,2,1}+t_{T,2,2}T+t_{T,2,3}T^{2}$ (S18)

To make the model more realistic, we considered the effect of water on CO_2_ adsorption for supported amines. This effect is described by the water effect factor *f*_water_ that has been shown to correlate with temperature, relative humidity, and CO_2_ partial pressure.[1, 3] Based on our CO_2_ adsorption tests, the following empirical equation is utilized to fit *f*_water_, thereby obtaining the CO_2_ adsorption capacity humid conditions:

$q_{\text{CO}_{2}\text{,humid}}=f_{\text{water}}\left(T,p_{\text{CO}_{2}},x_{\text{RH}}\right)q_{\text{CO}_{2}\text{,dry}}\left(T,p_{\text{CO}_{2}}\right)$ (S19)

$f_{\text{water}}=f_{T,\text{RH},1}p_{\text{CO}_{2}}^{f_{T,\text{RH},2}}$ (S20)

where *f_T_*_,RH,1_ and *f_T_*_,RH,2_ are parameters dependent on temperature and humidity, and they were fitted using the multiple linear regression method:

$f_{T,\text{RH},1}=f_{T,\text{RH},1,1}+f_{T,\text{RH},1,2}T+f_{T,\text{RH},1,3}x_{\text{RH}}$ (S21)

$f_{T,\text{RH},2}=f_{T,\text{RH},2,1}+f_{T,\text{RH},2,2}T+f_{T,\text{RH},2,3}x_{\text{RH}}$ (S22)

The fitted parameters of isotherm models can be found in Table S1.

The isosteric adsorption heat of CO_2_ and water are calculated using the isotherms at different temperatures by Clausius-Clapeyron equation and found to be stable at −60 kJ/mol and −42 kJ/mol, respectively.

The linear driving force (LDF) model is used to describe the adsorption/desorption kinetics of water and CO_2_[4]:

$\frac{\partial q_{i}}{\partial t}=k_{\text{LDF,}i}\left(q_{\text{e,}i}-q_{i}\right),\text{ }i\text{ = CO}_{2}\text{, H}_{2}\text{O}$ (S23)

(4) Additional conditions

The column pressure is calculated using the ideal gas law:

$p=C_{\text{T}}RT$ (S24)

The gas velocity inside the channel is described by the Hagen–Poiseuille equation[5]:

$v=-\frac{R_{\text{eq}}^{2}}{8\mu_{\text{gas}}}\frac{\partial p}{\partial z}$ (S25)

The condensation step is modeled assuming the difference between the bulk concentration of water vapor molecules and its concentration at the interface is the driving force for water condensation.[5] The mass transfer constant was calculated using the correlation given by Jeong et al. [6] The concentration at the interface *C*_s,H2O_ is assumed to be in equilibrium with pure liquid water and thus estimated using the Antoine equation[7]:

$rate_{\text{condense}}=k_{\text{condense}}\left(C_{\text{H}_{2}\text{O}}-C_{\text{H}_{2}\text{O,sat}}\right)$ (S26)

The adsorption section and desorption sections within the rotary adsorber are isolated by a dynamic sealing structure, hence they are not completely sealed. Gas leakage from the adsorption to the desorption section is considered to be proportional to the pressure difference between the two sections, with a leakage rate coefficient *k*_leak_ of 1.25×10^-6^ 1/Pa[8]:

$F_{\text{leak}}=k_{\text{leak}}F_{\text{air}}\left(p_{\text{adsorption section}}-p_{\text{desorption section}}\right)$ (S27)

The initial conditions and boundary conditions of the steam-purge rotary adsorber are listed in Table S2. The parameters of the baseline simulation group are shown in Table S3. In the parametric study, the parameters adjusted for the other groups are presented in Table S4.

Table S1 The values of parameters in the isotherm and kinetic models for AFHM.

| Symbol | Value |
| --- | --- |
| *c*_m,GAB_ | 2.56804 |
| *c*_G1_ | 1.683 |
| *c*_G2_ | -6255.469 |
| *c*_G3_ | 0.972 |
| *K*_ads1_ | 0.671 |
| *K*_ads2_ | -363172000 |
| *K*_ads3_ | 0.925 |
| *n*_s,_*_T_*_,1,1_ | 7.019 |
| *n*_s,_*_T_*_,1,2_ | -0.03164 |
| *n*_s,_*_T_*_,1,3_ | 3.49×10^-5^ |
| *b_T_*_,1,1_ | 0.01715 |
| *b_T_*_,1,2_ | -4.28×10^12^ |
| *b_T_*_,1,3_ | 0.90598 |
| *t_T_*_,1,1_ | 0.24023 |
| *t_T_*_,1,2_ | 2.53×10^-78^ |
| *t_T_*_,1,3_ | 30.31602 |
| *n*_s,_*_T_*_,2,1_ | 0.00288 |
| *n*_s,_*_T_*_,2,2_ | 215.86647 |
| *b_T_*_,2,1_ | 7.86×10^55^ |
| *b_T_*_,2,2_ | -22.18586 |
| *t_T_*_,2,1_ | -2.25834 |
| *t_T_*_,2,2_ | 0.02772 |
| *t_T_*_,2,3_ | -5.22×10^-5^ |
| *f_T_*_,RH,1,1_ | 3.21294 |
| *f_T_*_,RH,1,2_ | -0.00637 |
| *f_T_*_,RH,1,3_ | 1.20853 |
| *f_T_*_,RH,2,1_ | -0.0122 |
| *f_T_*_,RH,2,2_ | -2.06×10^-4^ |
| *f_T_*_,RH,3,3_ | -0.07076 |
| *k*_LDF,CO2_, when *q*≤*q*_e_ | 0.00143 |
| *k*_LDF,CO2_, when *q*>*q*_e_ | 0.00665 |
| *k*_LDF,H2O_, when *q*≤*q*_e_ | 0.00153 |
| *k*_LDF,H2O_, when *q*≤*q*_e_ | 0.00664 |

Table S2 The initial conditions and boundary conditions of the steam-purge rotary adsorber model.

| Initial conditions | |
| --- | --- |
| *C_i_*_,_*_t_*_=0_=*C_i_*_,air_, *T*_gas,_*_t_*_=0_=*T*_air_, *T*_sol,_*_t_*_=0_=*T*_air_ | |
| Boundary conditions | |
| Adsorption step | |
| Inlet, *z*=0 | Outlet, *z*=*L* |
| $D_{\text{ax},i}C_{\text{T}}\left(0\right)\frac{\partial x_{i}\left(0\right)}{\partial z}=v\left(0\right)C_{i}\left(0\right)-v_{\text{air}}C_{\text{air,}i}$ | $\frac{\partial C_{i}\left(L\right)}{\partial z}=0$ |
| $v\left(0\right)C_{\text{T}}\left(0\right)=v_{\text{air}}C_{\text{air,T}}$ | $\left\{\begin{matrix} v\left(L\right)=0,p\left(L\right)\leq p_{\text{air}} \\ \frac{\partial v\left(L\right)}{\partial z}=0,p\left(L\right)>p_{\text{air}} \end{matrix}\right.$ |
| $\frac{\partial p\left(0\right)}{\partial z}=-\frac{8\mu_{\text{air}}v\left(0\right)}{R_{1}^{2}}$ | $\left\{\begin{matrix} \frac{\partial p\left(L\right)}{\partial z}=-\frac{8\mu_{\text{air}}v\left(L\right)}{R_{1}^{2}},p\left(L\right)\leq p_{\text{air}} \\ p\left(L\right)=p_{\text{air}},p\left(L\right)>p_{\text{air}} \end{matrix}\right.$ |
| $\lambda_{\text{air}}\frac{\partial T_{\text{gas}}\left(0\right)}{\partial z}=\rho_{\text{air}}c_{\text{p,air}}v\left(0\right)T_{\text{gas}}\left(0\right)-\rho_{\text{air}}c_{\text{p,air}}v_{\text{air}}T_{\text{air}}$ | $\frac{\partial T_{\text{gas}}\left(L\right)}{\partial z}=0$ |
| $\frac{\partial T_{\text{sol}}\left(0\right)}{\partial z}=0$ | $\frac{\partial T_{\text{sol}}\left(L\right)}{\partial z}=0$ |
| Desorption step | |
| Inlet, *z*=0 | Outlet, *z*=*L* |
| $D_{\text{ax},i}C_{\text{T}}\left(0\right)\frac{\partial x_{i}\left(0\right)}{\partial z}=v\left(0\right)C_{i}\left(0\right)-v_{\text{steam}}C_{\text{steam,}i}$ | $\frac{\partial C_{i}\left(L\right)}{\partial z}=0$ |
| $v\left(0\right)C_{\text{T}}\left(0\right)=v_{\text{steam}}C_{\text{steam,T}}$ | $\left\{\begin{matrix} v\left(L\right)=0,p\left(L\right)\leq p_{\text{steam}} \\ \frac{\partial v\left(L\right)}{\partial z}=0,p\left(L\right)>p_{\text{steam}} \end{matrix}\right.$ |
| $\frac{\partial p\left(0\right)}{\partial z}=-\frac{8\mu_{\text{steam}}v\left(0\right)}{R_{1}^{2}}$ | $\left\{\begin{matrix} \frac{\partial p\left(L\right)}{\partial z}=-\frac{8\mu_{\text{steam}}v\left(L\right)}{R_{1}^{2}},p\left(L\right)\leq p_{\text{steam}} \\ p\left(L\right)=p_{\text{steam}},p\left(L\right)>p_{\text{steam}} \end{matrix}\right.$ |
| $\lambda_{\text{steam}}\frac{\partial T_{\text{gas}}\left(0\right)}{\partial z}=\rho_{\text{steam}}c_{\text{p,steam}}v\left(0\right)T_{\text{gas}}\left(0\right)-\rho_{\text{steam}}c_{\text{p,steam}}v_{\text{steam}}T_{\text{steam}}$ | $\frac{\partial T_{\text{gas}}\left(L\right)}{\partial z}=0$ |
| $\frac{\partial T_{\text{sol}}\left(0\right)}{\partial z}=0$ | $\frac{\partial T_{\text{sol}}\left(L\right)}{\partial z}=0$ |

Table S3 The values of parameters used for simulation study.

| Parameter | Value |
| --- | --- |
| Structural parameters | |
| *a* (mm) | 1 |
| *b* (mm) | 1.5 |
| *δ* (mm) | 0.25 |
| *L* (m) | 0.3 |
| *f*_gas_ | 0.816 |
| *S*_total_ | =2*a*∙*b* |
| *S*_gas_ | = *f*_gas_∙*S*_total_ |
| *P*_channel_ | $=2b+2\sqrt{b^{2}+\left(a\pi\right)^{2}}\frac{3+\left(2b/a\pi\right)^{2}}{4+\left(2b/a\pi\right)^{2}}$ |
| *As*_1_ (m) | =*S*_gas_/*P* |
| *As*_2_ (m) | =(*S*_total_ – *S*_gas_)/*P* |
| *ASAR* | 5/6 |
| *DSAR* | 1/6 |
| Operating parameters | |
| *T*_ambient_ (K) | 298.15 |
| *p*_ambient_ (bar) | 1.013 |
| *v*_air_ (m/s) | 1 |
| *p*_air_ (bar) | 1.013 |
| *x*_air_ | CO_2_: 0.04%, N_2_:77.38%, O_2_: 21.00%, H_2_O: 1.56% |
| *T*_air_ (K) | 298.15 |
| *v*_steam_ (m/s) | 0.01 |
| *p*_steam_ (bar) | 1.014 |
| *x*_steam_ | H_2_O: 100% |
| *T*_steam_ (K) | 378.18 |
| *n*_rot_ (r/h) | 1.67 |
| Physical and chemical properties | |
| *ρ*_ads_ (kg/m^3^) | 833 |
| *c*_p,ads_ (J/kg/K) | 930 |
| *λ*_ads_ (W/m/K) | 0.5089 |
| *h* (W/m^2^/K) | 75 |
| *D*_ax_ (m^2^/s) | 1.6×10^-5^ |
| *L*_H2O_ (J/mol) | 40800 |
| *∆H*_CO2_ (J/mol) | 60000 |
| *∆H*_H2O_ (J/mol) | 42000 |

# S3. Adsorbent characterization


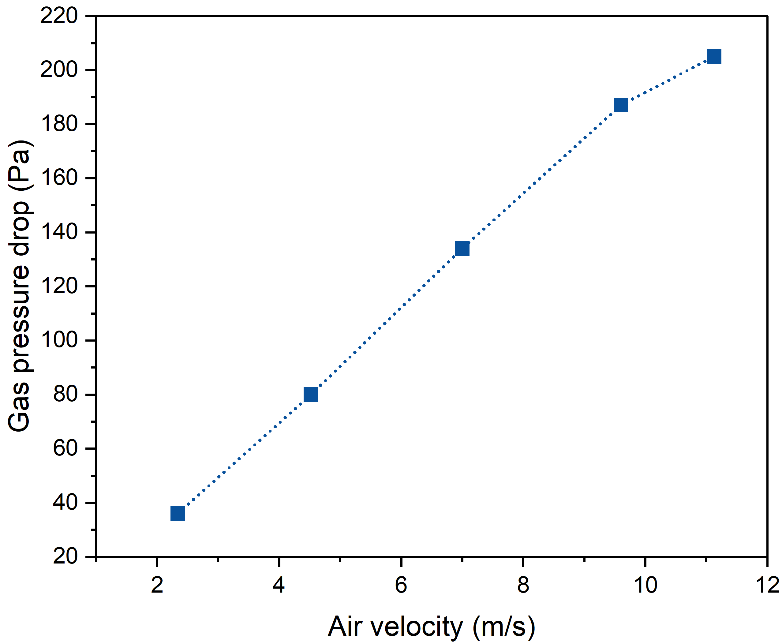


Figure S5 The gas pressure drop before and after passing through the structured adsorbent. The test was conducted using a rotor with a diameter of 800 mm and thickness of 300 mm at 25°C.

# S4. Prototype tests


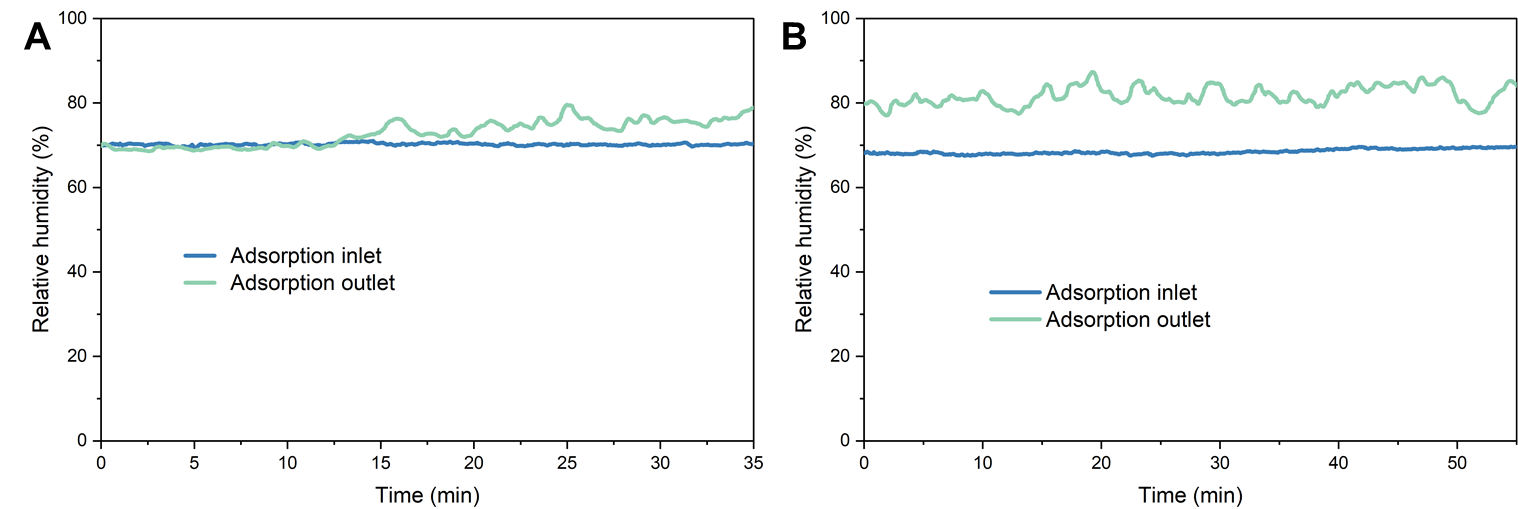


Figure S6 Relative humidities at the inlet and outlet of flow paths during (A) the unsteady state operation and (B) the steady state operation.


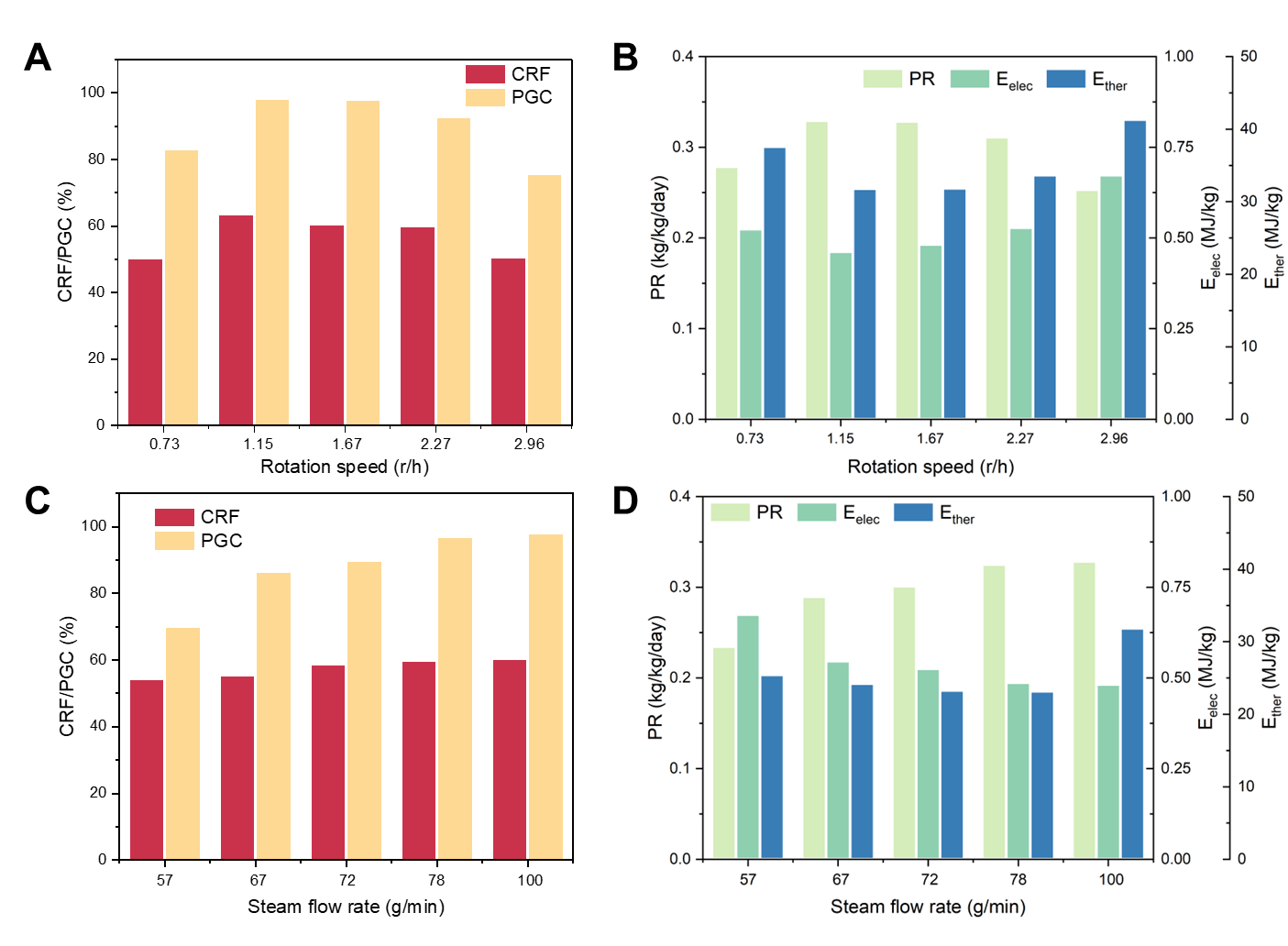


Figure S7 Performance of prototype under varied conditions. (A−B) Performance indicators at different rotation speeds. (C−D) Performance indicators under different steam flow rates.


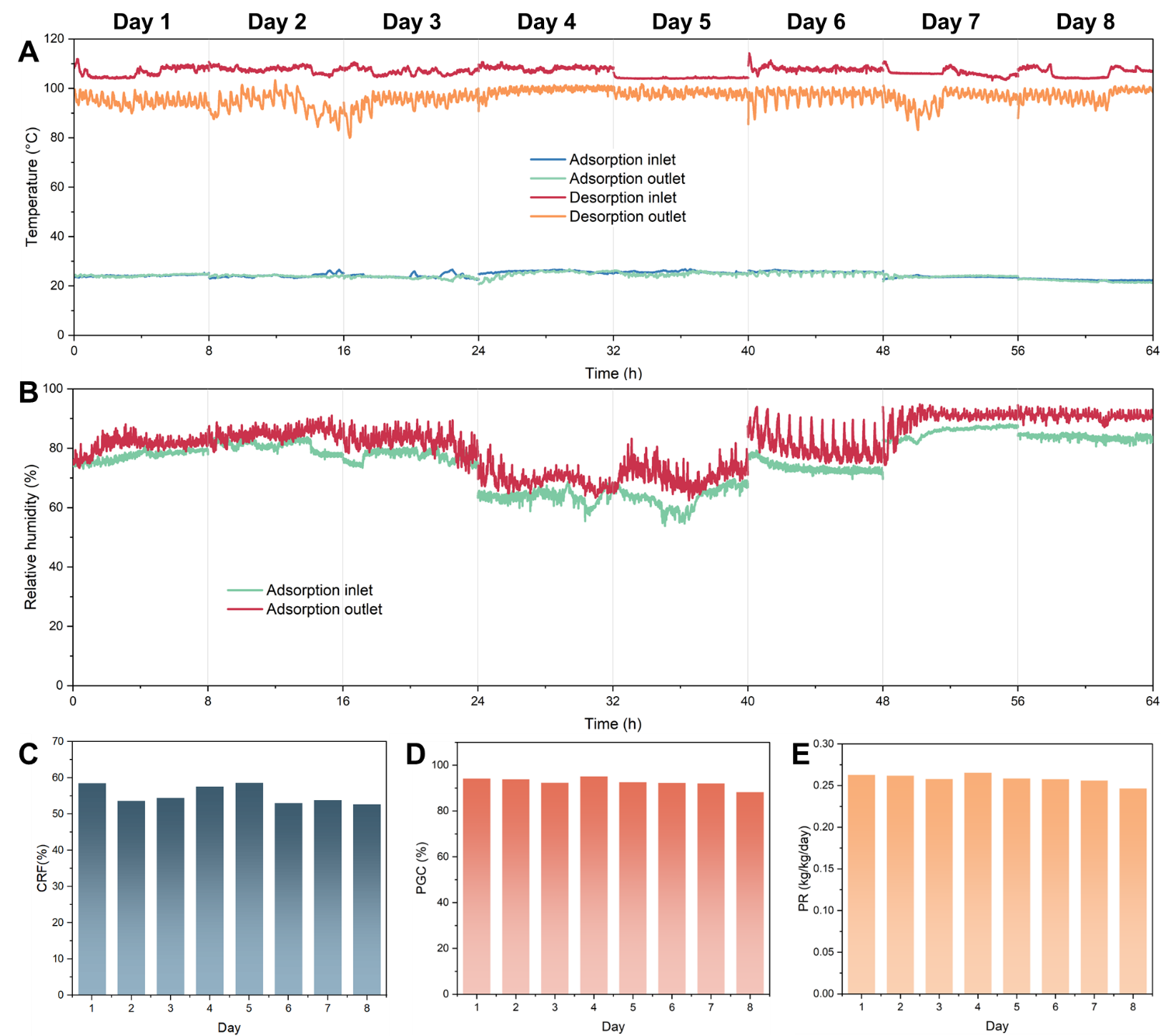


Figure S8 (A) The temperature variations during the eight-day tests. (B) The relative humidity variations during the eight-day tests. (C–E) The daily average *CRF*, *PGC*, and *PR* of the rotary adsorber during the eight-day tests.

# S5. Model validation


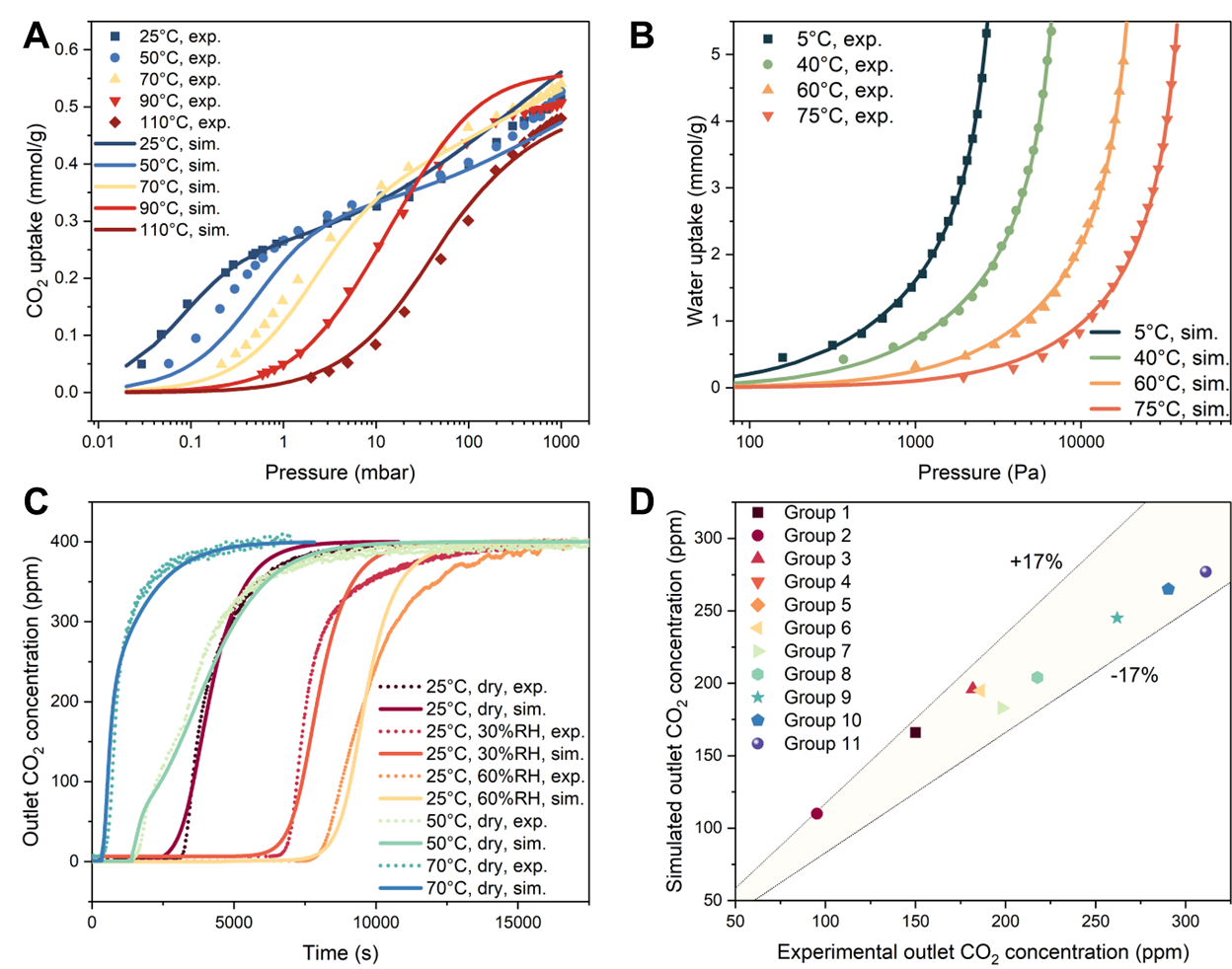


Figure S9 (A) The experimental and simulated CO_2_ isotherms under dry conditions. (B) The experimental and simulated water isotherms. (C) The experimental and simulated CO_2_ concentration in the fixed tests. (D) The experimental and simulated adsorption outlet CO_2_ concentration in the rotary adsorber prototypes tests.

# S6. Simulation parametric study


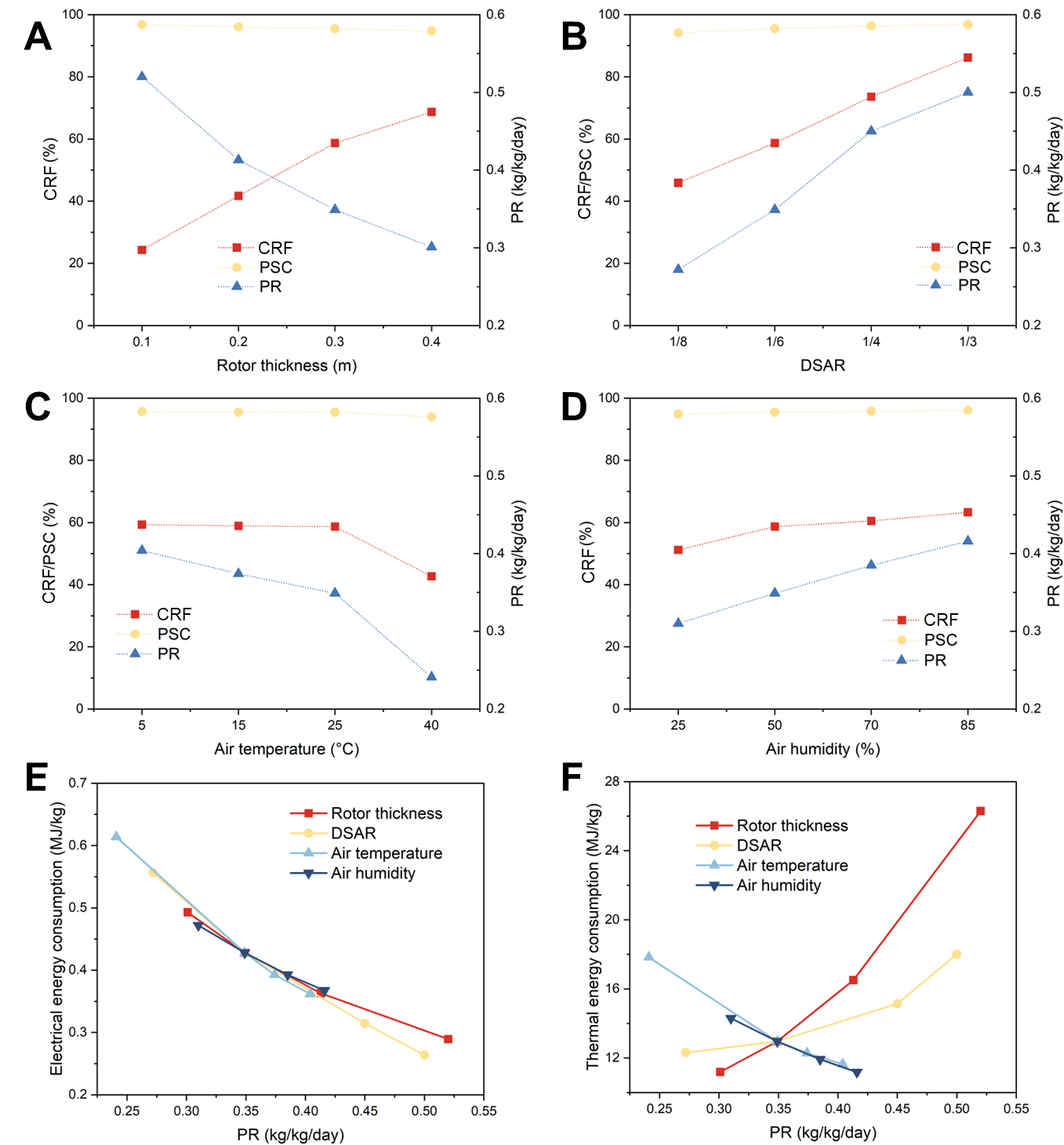


Figure S10 The parametric study in simulation investigating the effects of (A) rotor thickness, (B) *DSAR*, (C) air temperature, and (D) air humidity on *CRF*, *PGC* and *PR*. (E) The relationship between *PR* and *E*_elec_ when adjusting different parameters. (F) The relationship between *PR* and *E*_ther_ when adjusting different parameters.

Table S4 Effect of structural parameters and environmental parameters on the steam-purge rotary adsorber performance.

| Simulation | *L*  (m) | *DSAR*  (-) | *T*_air_  (°C) | *x*_RH,air_  (%) | *CRF*  (%) | *PGC*  (%) | *PR*  (kg/kg/day) | *E*_blower_  (MJ/kg_CO2_) | *E*_motor_  (MJ/kg_CO2_) | *E*_steam_  (MJ/kg_CO2_) |
| --- | --- | --- | --- | --- | --- | --- | --- | --- | --- | --- |
| 1* | 0.3 | 1/6 | 25 | 50 | 58.7 | 95.5 | 0.349 | 0.240 | 0.188 | 12.96 |
| 2 | 0.1 | 1/6 | 25 | 50 | 24.3 | 96.8 | 0.520 | 0.162 | 0.128 | 26.30 |
| 3 | 0.2 | 1/6 | 25 | 50 | 41.7 | 96.1 | 0.413 | 0.204 | 0.160 | 16.51 |
| 4 | 0.4 | 1/6 | 25 | 50 | 68.7 | 94.8 | 0.301 | 0.276 | 0.217 | 11.19 |
| 5 | 0.3 | 1/8 | 25 | 50 | 45.9 | 94.2 | 0.272 | 0.319 | 0.238 | 12.31 |
| 6 | 0.3 | 1/4 | 25 | 50 | 73.6 | 96.4 | 0.450 | 0.168 | 0.146 | 15.14 |
| 7 | 0.3 | 1/3 | 25 | 50 | 86.2 | 96.8 | 0.500 | 0.133 | 0.131 | 18.00 |
| 8 | 0.3 | 1/6 | 5 | 50 | 59.3 | 95.7 | 0.404 | 0.198 | 0.164 | 11.64 |
| 9 | 0.3 | 1/6 | 15 | 50 | 58.9 | 95.5 | 0.374 | 0.218 | 0.175 | 12.28 |
| 10 | 0.3 | 1/6 | 40 | 50 | 42.7 | 94.0 | 0.241 | 0.349 | 0.265 | 17.83 |
| 11 | 0.3 | 1/6 | 25 | 25 | 51.2 | 94.9 | 0.310 | 0.265 | 0.207 | 14.30 |
| 12 | 0.3 | 1/6 | 25 | 70 | 60.5 | 95.8 | 0.385 | 0.220 | 0.173 | 11.92 |
| 13 | 0.3 | 1/6 | 25 | 85 | 63.3 | 96.1 | 0.416 | 0.206 | 0.162 | 11.18 |

* It represents the baseline simulation group.

# S7. Optimization strategies

## S7.1 Heat recovery design


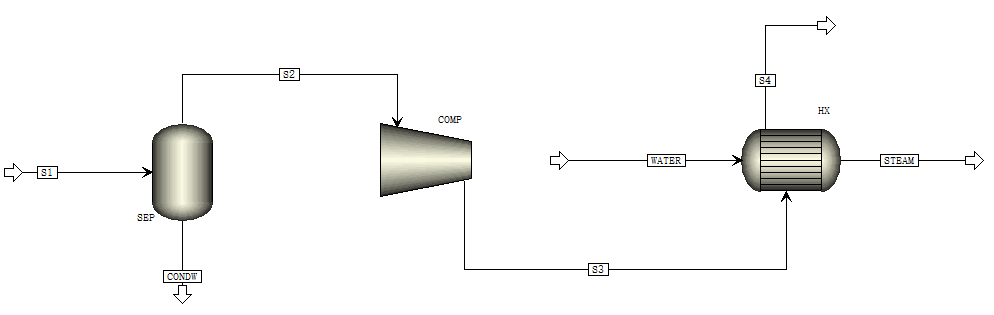


Figure S11 Heat recovery model in the Aspen Plus software.

## S7.2 Adsorbent enhancement


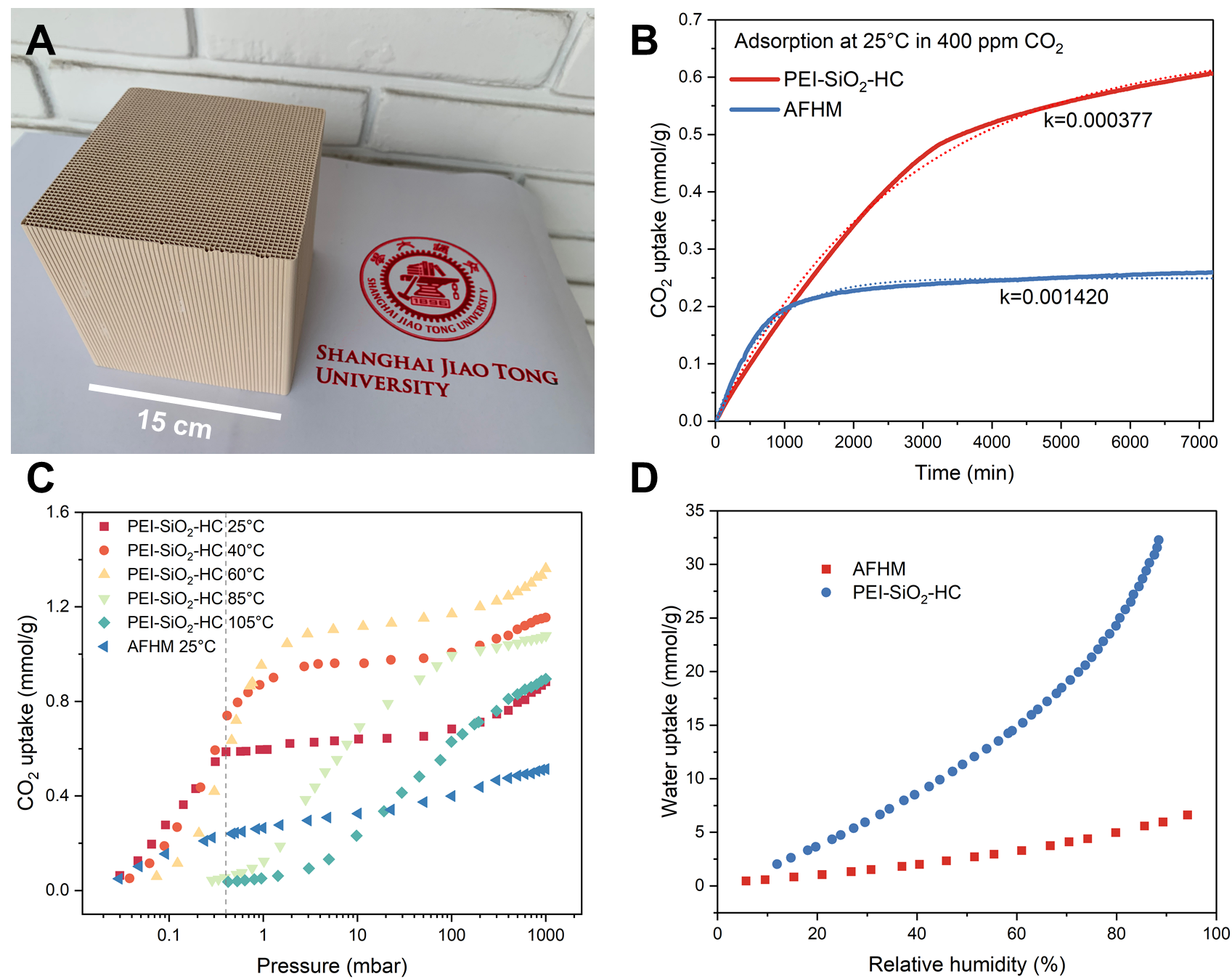


Figure S12 (A) The photograph of the substrate-free structured adsorbent PEI-SiO_2_-HC. (B) The dynamic adsorption curves. (C) The CO_2_ adsorption isotherms. (D) The water adsorption isotherms.

For the PEI-SiO_2_-HC used in the steam-purge rotary adsorber, its water adsorption isotherm is fitted using the temperature-dependent GAB model:

$q_{\text{H}_{2}\text{O}}=c_{\text{m,GAB}}\frac{c_{\text{G}}K_{\text{ads}}x_{\text{RH}}}{\left(1-K_{\text{ads}}x_{\text{RH}}\right)\left(1+\left(c_{\text{G}}-1\right)K_{\text{ads}}x_{\text{RH}}\right)}$ (S28)

For the PEI-SiO_2_-HC, its CO_2_ adsorption isotherm under the dry condition is fitted using the dual site Toth model:

$q_{\text{CO}_{2}\text{,dry}}=\frac{n_{\text{s,}T,1}b_{T,1}p_{\text{CO}_{2}}}{\left(1+\left(b_{T,1}p_{\text{CO}_{2}}\right)^{t_{T,1}}\right)^{1/t_{T,1}}}+\frac{n_{\text{s,}T,2}b_{T,2}p_{\text{CO}_{2}}}{\left(1+\left(b_{T,2}p_{\text{CO}_{2}}\right)^{t_{T,2}}\right)^{1/t_{T,2}}}$ (S29)

where *n*_s,_*_T_*_,1_, *b_T_*_,1_, *t_T_*_,1_, *n*_s,_*_T_*_,2_, *b_T_*_,2_, *t_T_*_,2_ are temperature-dependent parameters and fitted using the empirical formulas:

$n_{\text{s},T,1}=n_{\text{s},1,1}+n_{\text{s},1,2}T+n_{\text{s},1,3}T^{2}+n_{\text{s},1,4}T^{3}+n_{\text{s},1,5}T^{4}$ (S30)

$b_{T,1}=b_{1,1}-b_{1,2}b_{1,3}^{T}$ (S31)

$t_{T,1}=t_{1,1}+t_{1,2}T+t_{1,3}T^{2}+t_{1,4}T^{3}+t_{1,5}T^{4}$ (S32)

$n_{\text{s},T,2}=n_{\text{s},2,1}+n_{\text{s},2,2}T+n_{\text{s},2,3}T^{2}+n_{\text{s},2,4}T^{3}+n_{\text{s},2,5}T^{4}$ (S33)

$b_{T,2}=b_{2,1}\left(1+T\right)^{b_{2,2}}$ (S34)

$t_{T,2}=t_{2,1}+t_{2,2}T+t_{2,3}T^{2}+t_{2,4}T^{3}$ (S35)

The fitted parameters of isotherm models can be found in Table S5.

Table S5 The values of parameters in the isotherm and kinetic models for PEI-SiO_2_-HC.

| Symbol | Value |
| --- | --- |
| *c*_m,GAB_ | 11.98799 |
| *c*_G_ | 2.12903 |
| *K*_ads_ | 0.7779957 |
| *n*_s,1,1_ | 1728.29284 |
| *n*_s,1,2_ | -21.01976 |
| *n*_s,1,3_ | 0.09537 |
| *n*_s,1,4_ | -0.0001912 |
| *n*_s,1,5_ | 1.4298×10^-7^ |
| *b*_1,1_ | -0.15534 |
| *b*_1,2_ | -770329.05 |
| *b*_1,3_ | 0.96067 |
| *t*_1,1_ | 25564.74632 |
| *t*_1,2_ | -307.15217 |
| *t*_1,3_ | 1.37940 |
| *t*_1,4_ | -0.0027438 |
| *t*_1,5_ | 2.0396×10^-6^ |
| *n*_s,2,1_ | -5080.84265 |
| *n*_s,2,2_ | 60.63653 |
| *n*_s,2,3_ | -0.27034 |
| *n*_s,2,4_ | 0.000533714 |
| *n*_s,2,5_ | -3.93741×10^-7^ |
| *b*_2,1_ | 2.06961×10^31^ |
| *b*_2,2_ | -12.66318 |
| *t*_2,1_ | 136.30972 |
| *t*_2,2_ | -1.26380477 |
| *t*_2,3_ | 0.003873226 |
| *t*_2,4_ | -3.91166×10^-6^ |
| *f_T_*_,RH,1,1_ | 3.21294 |
| *f_T_*_,RH,1,2_ | -0.00637 |
| *f_T_*_,RH,1,3_ | 1.20853 |
| *f_T_*_,RH,2,1_ | -0.0122 |
| *f_T_*_,RH,2,2_ | -2.06×10^-4^ |
| *f_T_*_,RH,3,3_ | -0.07076 |
| *k*_LDF,CO2_, when *q*≤*q*_e_ | 0.000377 |
| *k*_LDF,CO2_, when *q*>*q*_e_ | 0.00356 |
| *k*_LDF,H2O_, when *q*≤*q*_e_ | 0.00153 |
| *k*_LDF,H2O_, when *q*≤*q*_e_ | 0.00664 |

Table S6 The optimization groups for the rotary adsorber.

| Simulation | *L*  (m) | *DSAR*  (-) | *T*_air_  (°C) | *x*_RH,air_  (%) | *n*_rot_  (r/h) | *CRF*  (%) | *PGC*  (%) | *PR*  (kg/kg/day) | *T*_des,out_  (°C) | *x*_des,out,CO2_  (%) | *x*_des,out,N2_  (%) | *x*_des,out,O2_  (%) | *x*_des,out,H2O_  (%) | *E*_blower_  (MJ/kg) | *E*_motor_  (MJ/kg) | *E*_comp_  (MJ/kg) | *E*_ther_  (MJ/kg) | *E*_ther,rec_  (MJ/kg) |
| --- | --- | --- | --- | --- | --- | --- | --- | --- | --- | --- | --- | --- | --- | --- | --- | --- | --- | --- |
| HR1 | 0.3 | 1/6 | 15 | 25 | 1.67 | 76.5 | 95.6 | 0.271 | 91.4 | 8.39 | 0.18 | 0.05 | 91.39 | 0.181 | 0.150 | 0.228 | 11.68 | 8.86 |
| HR2 | 0.3 | 1/6 | 25 | 25 | 1.67 | 51.2 | 94.9 | 0.310 | 96.3 | 9.88 | 0.30 | 0.08 | 89.74 | 0.241 | 0.188 | 0.873 | 14.30 | 6.11 |
| HR3 | 0.3 | 1/6 | 25 | 50 | 1.67 | 58.7 | 95.5 | 0.349 | 93.7 | 9.52 | 0.22 | 0.06 | 90.20 | 0.218 | 0.171 | 0.544 | 12.96 | 8.71 |
| HR4 | 0.3 | 1/6 | 25 | 70 | 1.67 | 63.3 | 96.1 | 0.416 | 90.6 | 8.62 | 0.16 | 0.04 | 91.18 | 0.187 | 0.147 | 0.390 | 11.18 | 8.84 |
| HR5 | 0.3 | 1/6 | 35 | 70 | 1.67 | 51.2 | 95.1 | 0.300 | 96.1 | 7.63 | 0.18 | 0.05 | 92.14 | 0.250 | 0.193 | 0.822 | 14.41 | 6.89 |
| HRAE1 | 0.3 | 1/8 | 15 | 25 | 1 | 97.9 | 98.8 | 0.865 | 97.7 | 38.80 | 0.26 | 0.07 | 60.87 | 0.095 | 0.042 | 0.288 | 4.21 | 2.71 |
| HRAE2 | 0.3 | 1/8 | 25 | 25 | 1 | 98.7 | 98.9 | 0.920 | 96.7 | 36.78 | 0.27 | 0.07 | 62.88 | 0.089 | 0.039 | 0.312 | 3.76 | 2.06 |
| HRAE3 | 0.3 | 1/8 | 25 | 50 | 1 | 95.3 | 98.7 | 0.800 | 90.1 | 28.94 | 0.15 | 0.04 | 70.87 | 0.106 | 0.046 | 0.384 | 4.50 | 2.12 |
| HRAE4 | 0.3 | 1/8 | 25 | 70 | 1 | 92.8 | 98.8 | 0.853 | 89.8 | 23.40 | 0.09 | 0.02 | 76.49 | 0.102 | 0.045 | 0.373 | 4.36 | 2.14 |
| HRAE5 | 0.3 | 1/8 | 35 | 70 | 1 | 90.4 | 98.9 | 0.880 | 89.2 | 22.89 | 0.09 | 0.02 | 77.00 | 0.102 | 0.043 | 0.358 | 4.20 | 2.16 |

# S8. References

1. Wurzbacher, J.A., et al., Heat and mass transfer of temperature–vacuum swing desorption for CO_2_ capture from air*.* *Chemical Engineering Journal*, 2016, 283: 1329-1338.

2. Son, K.N., et al., Measurement and Prediction of the Heat of Adsorption and Equilibrium Concentration of CO2 on Zeolite 13X*.* *Journal of Chemical & Engineering Data*, 2018, 63(5): 1663-1674.

3. Young, J., et al., The impact of binary water–CO_2_ isotherm models on the optimal performance of sorbent-based direct air capture processes*.* *Energy & Environmental Science*, 2021, 14: 5377-5394.

4. Wang, Y., D. Zhao, and G.K. Li, Temperature-dependent kinetic analysis of direct air capture using a gravimetric approach in porous environments*.* *Adsorption*, 2025, 31(4).

5. Sinha, A., et al., Systems design and economic analysis of direct air capture of CO_2_ through temperature vacuum swing adsorption using MIL-101(Cr)-PEI-800 and mmen-Mg_2_(dobpdc) MOF adsorbents*.* *Industrial & Engineering Chemistry Research*, 2017, 56(3): 750-764.

6. Jeong, K., et al., Analytical modeling of water condensation in condensing heat exchanger*.* *International Journal of Heat and Mass Transfer*, 2010, 53(11): 2361-2368.

7. Huang, J., A Simple Accurate Formula for Calculating Saturation Vapor Pressure of Water and Ice*.* *Journal of Applied Meteorology and Climatology*, 2018, 57(6): 1265-1272.

8. M.Sc., E.P., A Kinetic Model of a CO_2_ Recycling Rotary Adsorption Wheel for Gas Turbine Power Plants with Carbon Capture. 2019, The University of Edinburgh.
